# Changes in the burden and underlying causes of heart failure in the Eastern Mediterranean Region, 1990–2019: An analysis of the Global Burden of Disease Study 2019

**DOI:** 10.1016/j.eclinm.2022.101788

**Published:** 2022-12-26

**Authors:** Razieh Hassannejad, Davood Shafie, Karam I. Turk-Adawi, Ahmad Mohammad Hajaj, Kamran Mehrabani-Zeinabad, Michelle Lui, Jacek A. Kopec, Hanan F. Abdul Rahim, Saeid Safiri, Ibtihal Fadhil, Wagida A. Anwar, Ali H. Mokdad, Sheikh Mohammed Shariful Islam, Nizal Sarrafzadegan

**Affiliations:** a Isfahan Cardiovascular Research Center, Cardiovascular Research Institute, Isfahan University of Medical Sciences, Isfahan, Iran; b Heart Failure Research Center, Cardiovascular Research Institute, Isfahan University of Medical Sciences, Isfahan, Iran; c Department of Public Health, College of Health Sciences, QU Health, Qatar University, Doha, Qatar; d QU Health Research and Graduate Studies, QU Health, Qatar University, Doha, Qatar; e Cardiac Rehabilitation Research Center, Cardiovascular Research Institute, Isfahan University of Medical Sciences, Isfahan, Iran; f Faculty of Medicine, School of Population & Public Health, University of British Columbia, Vancouver, Canada; g Arthritis Research Canada, Vancouver, Canada; h Research Center for Integrative Medicine in Aging, Aging Research Institute, Tabriz University of Medical Sciences, Tabriz, Iran; i Department of Community Medicine, Faculty of Medicine, Tabriz University of Medical Sciences, Tabriz, Iran; j Eastern Mediterranean NCD Alliance, Dubai, United Arab Emirates; k Community Medicine Department, Faculty of Medicine, Ain Shams University, Egypt; l Armed Forces College of Medicine (AFCM), Egypt; m Institute for Health Metrics and Evaluation, University of Washington, Seattle, USA; n Department of Health Metrics Sciences, University of Washington, Seattle, USA; o Institute for Physical Activity and Nutrition, Deakin University, Melbourne, Australia

**Keywords:** Global burden of disease, Heart failure, Prevalence, Year lived with disability, Ischemic heart disease, Hypertension, Eastern mediterranean region

## Abstract

**Background:**

The burden of heart failure (HF) is high globally, but information on its burden in the Eastern Mediterranean Region (EMR) is limited. This study provides a systematic analysis of the burden and underlying causes of HF in the EMR, including at the country level, between 1990 and 2019.

**Methods:**

We used the 2019 Global Burden of Disease (GBD) data for estimates of prevalence, years lived with disability (YLDs), and underlying causes of HF in the EMR. Age-standardised prevalence, YLDs, and underlying causes of HF were compared by 5-year age groups (considering 15 years old and more), sex (male and female), and countries.

**Findings:**

In contrast with the decreasing trend of HF burden globally, EMR showed an increasing trend. Globally, the HF age-standardised prevalence and YLDs decreased by 7.06% (95% UI: −7.22%, −6.9%) and 6.82% (95% UI: −6.98%, −6.66%) respectively, from 1990 to 2019. The HF age-standardised prevalence and YLDs in the EMR in 2019 were 706.43 (95% UI: 558.22–887.87) and 63.46 (95% UI: 39.82–92.59) per 100,000 persons, representing an increase of 8.07% (95% UI: 7.9%, 8.24%) and 8.79% (95% UI: 8.61%, 8.97%) from 1990, respectively. Amongst EMR countries, the age-standardised prevalence and YLDs were highest in Kuwait, while Pakistan consistently had the lowest HF burden. The dramatic increase of the age-standardised prevalence and YLDs were seen in Oman (28.79%; 95% UI: 28.51%, 29.07% and 29.56%; 95% UI: 29.28%, 29.84%), while Bahrain witnessed a reduction over the period shown (−9.66%; 95% UI: −9.84%, −9.48% and−9.14%; 95% UI: −9.32%, −8.96%). There were significant country-specific differences in trends of HF burden from 1990 to 2019. Males had relatively higher rates than females in all age groups. Among all causes of HF in 2019, ischemic heart disease accounted for the highest age-standardised prevalence and YLDs, followed by hypertensive heart disease.

**Interpretation:**

The burden of HF in the EMR was higher than the global, with increasing age-standardised prevalence and YLDs in countries of the region. A more comprehensive approach is needed to prevent underlying causes and improve medical care to control the burden of HF in the region.

**Funding:**

None.

**Research in context**

**Evidence before this study**

We searched the databases Web of Science, ProQuest, PubMed and Scopus, E-journals Sage, Science Direct, Springer, Wiley, and other Non-Indexed Citations such as scientific overview, profile and reporting with the search terms “global burden of disease”, “heart failure”, “prevalence”, “year lived with disability”, “Eastern Mediterranean Region”, “burden of heart failure”, for articles published in English or any other languages of EMR countries from database inception to Jul 5, 2022. The identified scientific studies were very limited since in most EMR countries there is lack of data collections and statistics of disease, especially in the countries who suffered wars and migration in the past decade, where actually any form of statistics would be not available. The burden of heart failure (HF) and underlying causes in 195 countries and territories has been published from the Global Burden of Disease (GBD) study 2017, reporting prevalence, years lived with disability (YLDs), and underlying causes of HF between 1990 and 2017. To our knowledge, there have been no systematic overviews of gradients of HF burden and underlying causes across the EMR counties based on GBD 2019.

**Added value of this study**

Since EMR countries are burdened with conflicts and political instability that affect several countries’ infrastructure including health services, in this article, which is based on data from GBD 2019, we provided a comprehensive overview of the changes in the burden and underlying causes of HF between 1990 and 2019 in the EMR and its countries and compared by age, sex, SDI, Healthcare Access and Quality (HAQ) Index and location. Our results show an increase in the age-standardised prevalence and YLD rates in EMR during 1990–2019. There was also no substantial difference in the etiology spectrum of HF across the 22 countries. Overall, ischemic heart disease and hypertensive heart disease were the top causes of HF in the EMR. It was found that the highest observed HAQ Index values increased by a rise in SDI. There is a significant location and SDI or HAQ variation in the levels and trends of HF burden from 1990 to 2019.

**Implications of all the available evidence**

This study provides information on the burden of HF in the EMR and its countries. Awareness of HF burden in EMR informs the health decision-makers so that they should monitor the situation and strengthen efforts to designing and conducting cost-effective strategies based on early diagnosis and prevention of the underlying causes of HF to reduce its future burden in the region. In addition, these findings could be useful in the implementation of guidelines in the areas of medication and medical service accessibility, clinical practice patterns, and geographically specific public health policy, as well as tackling other hazards threatening the region's public health such as inequality (social, political, economic and gender), and conflicts and war.

## Introduction

The Eastern Mediterranean Region (EMR) comprises 22 countries with a total estimated population of 600 million individuals. The region is characterised by a young population, with 40% of its total population below the age of 20.^1^ The region is diversified in economic, social and health measures religion, and language. The region is burdened with conflicts and political instability that affect several countries’ infrastructure, including health services.

Heart failure (HF) is a major global health concern affecting about 26 million people worldwide.^2^^,^^3^ Clinically, HF is a highly complex syndrome resulting from an impaired heart function, mainly characterised by breathlessness, fluid retention, and fatigue.^4^^,^^5^

HF imposes a significant burden on health systems and is associated with substantially high prevalence and mortality rates. The International Congestive Heart Failure (INTER-CHF) study assessed 1-year-mortality outcomes for HF, globally at 16.5%, with highest rates in Africa (34%), followed by India (23%); and lowest in China (7%), South America (9%), and the Middle East (9%).^3^ However, this study considered only 108 centres in 16 countries include Africa (Mozambique, Nigeria, South Africa, Sudan, and Uganda), China, India, the Middle East (Egypt, Qatar, and Saudi Arabia), southeast Asia (Malaysia, and the Philippines), and South America (Argentina, Chile, Colombia, and Ecuador). Variations in mortality between regions could be the result of health-care infrastructure, quality and access, or environmental and genetic factors. For instance, it can be underestimated in the Middle East since only three Middle East countries with a high level of Socio-demographic Index (SDI) and Healthcare Access and Quality (HAQ) index were considered. Further studies in large, global cohorts are needed.

It has also been estimated that there were over 54 million cases with HF worldwide in 2018, and this number is expected to increase continuously during the next few decades.^6^ While population aging drives the increase in the overall incidence of HF,^2^^,^^7^^,^^8^ the age-adjusted incidence has been reported to be stable or even decreasing in several studies, particularly in developed countries.^2^ The increase in prevalence could be due to aging, whereas the reduction in incidence could be related to better management of cardiovascular diseases (CVD) and improved HF survival due to advancements in medical therapy and device-assisted technology.

Although the outcomes of HF have improved significantly due to the implementation of management programs, the implications of HF are still substantial.^4^ Apart from the human burden, the financial burden of HF-related expenditures is substantially high, at an estimated annual cost of US$108 billion globally.^9^ Population-based epidemiological studies of HF are needed, especially in low- and middle-income countries, to inform public health policy as well as health care delivery planning. In those countries, prevalence is projected to rise as populations age and the burden of HF risk factors, such as hypertension (HTN) and diabetes mellitus (DM), increases.^10^ A detailed understanding of the drivers of HF rates is also needed, as they vary extensively.^4^ For example, while hypertensive heart disease is reported to be the major cause of HF in Africa, ischemic heart disease (IHD) is more important prominent in East Asia.^11^^,^^12^

There are significant differences in the prevalence of HF, access to health care, and health policies both within countries of the EMR and other world regions.^13^, ^14^, ^15^ An epidemiological transition drives a surge in the prevalence of etiological factors, such as HTN, DM, and IHD. A recent 2017 Global Burden of Disease (GBD) study of HF reported a global age-standardised prevalence and years lived with disability (YLD) rates of 831.0 and 128.2 per 100,000 persons, respectively, representing a respective decrease of 7.2% and 0.9% from 1990. Among all causes of HF, IHD (26.5%) and HTN (26.2%) accounted for the highest proportion of age-standardised prevalence.

However, studies focusing on the burden of HF in the EMR countries are inadequate. The limited data on HF in the EMR may be attributed partially to the low number of population-based studies in this region. Therefore, we aimed to summarise recent data on HF in the EMR countries to develop adequate knowledge translation strategies and to identify research gaps for future focus. Specifically, we analysed available data on the prevalence, YLDs, and underlying causes of HF using the GBD data in the EMR region from 1990 to 2019 and provided comparisons within the region as well as with the global profile.

## Methods

Common strategies utilised for the GBD 2019 study have been detailed previously.^16^ GBD 2019, implemented by the Institute of Health Metrics and Evaluation (IHME), is the most comprehensive source of up-to-date evaluation and estimation of the burden of diseases and injuries for 204 countries and territories from 1990 to 2019. It provides data on trends of the most important causes of morbidity and mortality burden at global, regional, and national levels by age and sex. All estimates used and reported in this study are available publicly from the IHME website https://vizhub.healthdata.org/gbd-compare/and http://ghdx.healthdata.org/gbd-results-tool.

In the current study, the definition of the EMR is based on the World Health Organization (WHO) classification, which includes 22 countries: Afghanistan, Bahrain, Djibouti, Egypt, Iran, Iraq, Jordan, Kuwait, Lebanon, Libya, Morocco, Oman, Palestine, Pakistan, Qatar, Saudi Arabia, Somalia, Sudan, Syria, Tunisia, the United Arab Emirates (UAE), and Yemen.

HF was considered as an impairment in GBD data source. For GBD 2019, as in GBD 2017 and GBD 2016, the prevalence of HF impairment was estimated by country, age, sex, and year. Impairments in GBD are conditions or specific domains of functional health loss that are spread across many GBD causes as consequence. DisMod-MR 2.1 was utilised to estimate the overall impairment prevalence.^16^

### Heart failure impairment

#### Case definition and input data

Structured criteria such as the Framingham or European Society of Cardiology criteria were used for the clinical diagnosis of HF in GBD 2019. American College of Cardiology (ACC)/American Heart Association (AHA) Stage C and above was applied to capture both currently symptomatic persons and those diagnosed with HF but are currently asymptomatic. The burden of HF estimation process was done based on extracting literature data, inpatient hospital data, and claims data (supplementary, data source).^16^ More details about original data sources used for the estimations of HF can be found on the GBD 2019 Data Input Sources Tool website (http://ghdx.healthdata.org/gbd-2019/data-input-sources).

#### Modelling strategy and burden estimation

The etiologies cause of HF in this study were selected based on literature review and expert opinion including IHD, hypertensive heart disease, non-rheumatic valvular heart disease, rheumatic heart disease and alcoholic cardiomyopathy. The burden of HF was estimated based on the estimation of the overall prevalence of HF and then the proportion of HF attributable to each cause. The methods of estimation were explained in detail previously.

Bayesian meta-regression approach developed for GBD analyses through DisMod-MR 2.1 was applied to estimate the overall prevalence of HF. Estimates of the prevalence of HF due to Chagas, degenerative mitral valve disease, and calcific aortic valve disease were generated separately as part of the modelling strategy for those causes. Finally, an adjusted prevalence of HF due to all other etiologies was obtained by subtracting the prevalence of HF due to these causes from the overall HF estimates.^16^

To estimate the YLDs for HF, HF was first grouped into four severity levels, including treated, mild, moderate, and severe HF. Then, an empirically derived disability weight, which indicates the degree of health loss associated with the severity level on a scale from 0 (full health) to 1 (death), was assigned to each severity level of HF. Finally, model-based prevalence estimates at each severity level based on an analysis of Medical Expenditure Panel Surveys (MEPS) data, in combination with the corresponding disability weights, were used to calculate YLDs of HF for each age, sex, location, and year.^16^

Information about incidence and mortality from HF was not available and we only investigated prevalence and YLDs.

Consistent with the previous GBD studies to account for uncertainty, the 95% uncertainty intervals (UIs) were reported for each estimate. Uncertainty intervals were calculated by taking 1000 samples at each step of the calculation process from the posterior distribution of each quantity, and taking the 25th and 75th values of the ordered draws of the uncertainty distribution.^16^

For further evaluation, we used the SDI and HAQ Index, the indices created by GBD researchers. SDI ranged from 0.08 in Somalia to 0.88 in United Arab Emirates in 2019 and HAQ index varies between 19 in Somalia and 85.6 in Lebanon in 2016.

### Role of the funding source

There was no funding source for this study. RH and NS had access to all the data in the study, and had final responsibility for the decision to submit for publication.

## Results

### Regional-level estimates

**Fig. 1:**
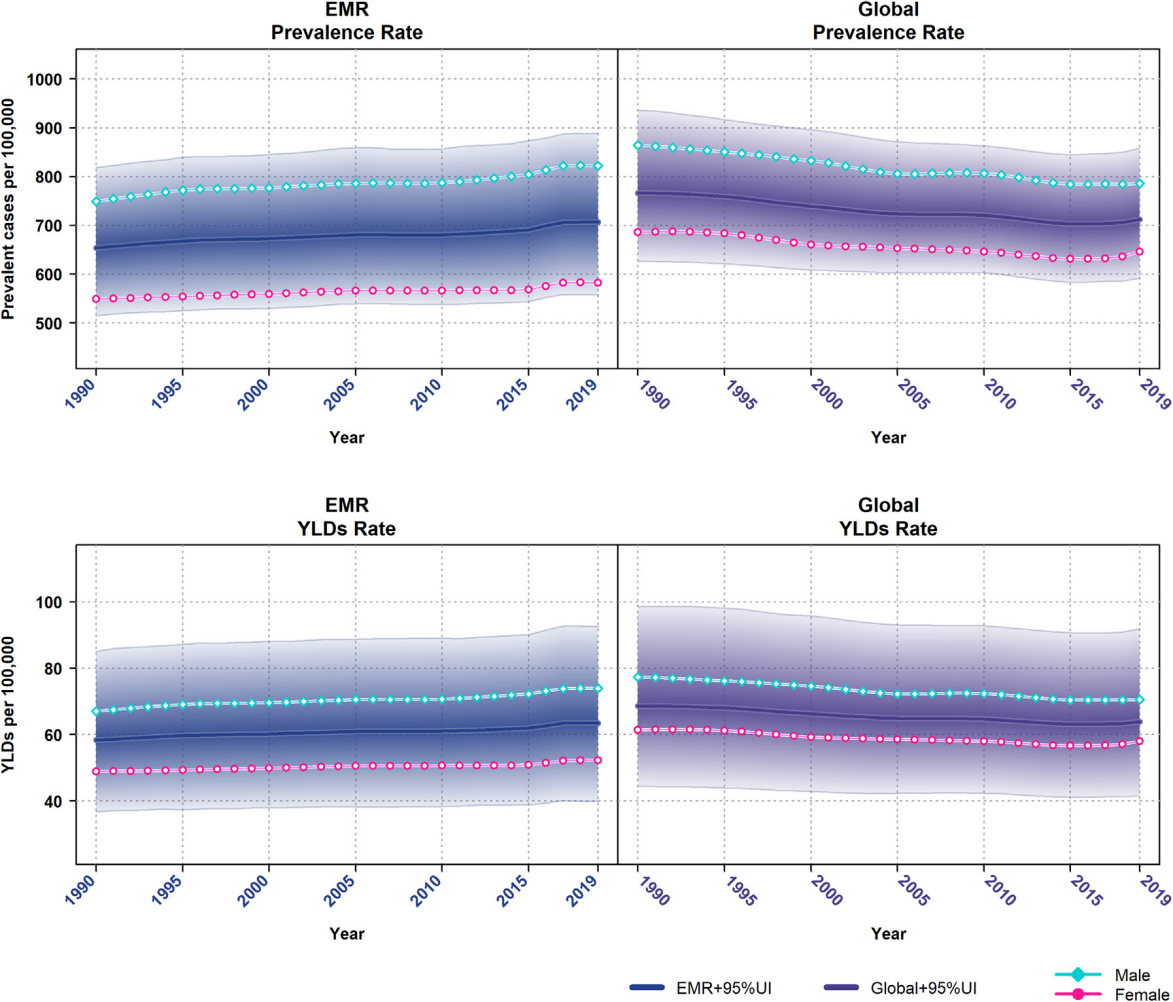
**Global and EMR age-standardised prevalence and YLD rates of heart failure (per 100,000 persons) for males and females, 1990–2019**. Shaded sections indicate 95% uncertainty intervals. YLD, years lived with disability; EMR, Eastern Mediterranean Region.

In 2019, the total number of cases with HF in the EMR was 2.76 million (95% UI: 2.18–3.45), of which 1.67 million (95% UI: 1.31–2.09) were male and 1.09 million (95% UI: 0.87–1.37) were female. The age-standardised prevalence rate was 706.43 per 100,000 persons (95% UI: 558.22–887.87); 822.27 (95% UI: 646.51–1044.66) for males and 582.58 (95% UI: 459.9–729.57) for females. Between 1990 and 2019, despite the decrease in age-standardised HF prevalence by 7.06% (95% UI: −7.22%, −6.9%) globally, from 765.99 (95% UI: 626.29–936.03) in 1990 to 711.90 (95% UI: 591.14–858.29) in 2019, there was an increase of 8.07% (95% UI: 7.9%, 8.24%) in the EMR, from 653.66 (95% UI: 514.94–818.58) per 100,000 persons in 1990 to 706.43 (95% UI: 558.22–887.87) in 2019. These opposing global and EMR trends persisted in 1990–2005 (−5.55%; 95% UI: −5.69%, −5.41% and 4.09%; 95% UI: 3.97%, 4.21%) and 2005–2019 (−1.6%; 95% UI: −1.68%, −1.52% and 3.82%; 95% UI: 3.7%, 3.94%) (Table 1 and Fig. 1). The trend was similar for males and females (Supplementary material Tables S1 and S2 and Fig. 1).

**Table 1:** Age-standardised prevalence rate (per 100,000 persons) of heart failure in 1990, 2005 and 2019, and their relative percentage change by EMR countries.

		Prevalence rate			%Δ		
		1990	2005	2019	1990–2005	2005–2019	1990–2019
	Global	765.99 (626.29–936.03)	723.49 (603.33–871.35)	711.90 (591.14–858.29)	−5.55 (−5.69, −5.41)	−1.6 (−1.68, −1.52)	−7.06 (−7.22, −6.9)
	EMR	653.66 (514.94–818.58)	680.41 (539.15–859.19)	706.43 (558.22–887.87)	4.09 (3.97, 4.21)	3.82 (3.7, 3.94)	8.07 (7.9, 8.24)
High SDI	Qatar	866.06 (719.44–1030.93)	954.87 (798.83–1126.59)	833.30 (638.42–1058.60)	10.25 (10.06, 10.44)	−12.73 (−12.94, −12.52)	−3.78 (−3.9, −3.66)
	United Arab Emirates	798.02 (619.96–1025.34)	770.17 (591.61–1005.54)	868.71 (663.43–1124.10)	−3.49 (−3.6, −3.38)	12.79 (12.58, 13)	8.86 (8.68, 9.04)
	Kuwait	977.51 (758.93–1243.27)	1031.22 (803.30–1329.26)	995.36 (772.49–1279.59)	5.49 (5.35, 5.63)	−3.48 (−3.59, −3.37)	1.83 (1.75, 1.91)
High-middle SDI	Oman	596.88 (493.83–724.27)	737.56 (568.88–942.81)	768.73 (598.08–969.18)	23.57 (23.31, 23.83)	4.23 (4.11, 4.35)	28.79 (28.51, 29.07)
	Saudi Arabia	644.90 (495.51–838.69)	750.04 (578.59–953.12)	780.11 (602.42–995.74)	16.3 (16.07, 16.53)	4.01 (3.89, 4.13)	20.97 (20.72, 21.22)
	Bahrain	865.08 (668.30–1099.09)	781.63 (602.30–993.45)	781.52 (603.84–996.54)	−9.65 (−9.83, −9.47)	−0.01 (−0.02, 0)	−9.66 (−9.84, −9.48)
	Lebanon	826.61 (637.08–1063.69)	836.25 (648.90–1079.88)	891.04 (686.61–1141.60)	1.17 (1.1, 1.24)	6.55 (6.4, 6.7)	7.79 (7.62, 7.96)
	Libya	791.84 (616.87–1009.03)	889.22 (687.21–1142.13)	892.44 (684.45–1153.09)	12.3 (12.1, 12.5)	0.36 (0.32, 0.4)	12.7 (12.49, 12.91)
	Jordan	934.48 (729.48–1208.37)	939.71 (734.37–1215.65)	951.92 (739.76–1225.12)	0.56 (0.51, 0.61)	1.3 (1.23, 1.37)	1.87 (1.79, 1.95)
Middle SDI	Egypt	717.42 (524.26–945.96)	712.41 (523.30–958)	766.01 (564.38–1021.33)	−0.7 (−0.75, −0.65)	7.52 (7.36, 7.68)	6.77 (6.61, 6.93)
	Syrian Arab Republic	699.44 (534.25–893.47)	739.73 (566.60–933.93)	769.42 (590.27–985.50)	5.76 (5.62, 5.9)	4.01 (3.89, 4.13)	10.01 (9.82, 10.2)
	Iraq	841.88 (641.98–1079.36)	792.41 (606.79–1017.11)	807.62 (615.96–1044.42)	−5.88 (−6.03, −5.73)	1.92 (1.83, 2.01)	−4.07 (−4.19, −3.95)
	Tunisia	802.94 (615.85–1033.55)	814.44 (625.35–1042.45)	825.94 (643.16–1057.13)	1.43 (1.36, 1.5)	1.41 (1.34, 1.48)	2.86 (2.76, 2.96)
	Iran (Islamic Republic of)	926.63 (735.05–1154.11)	898.44 (714.10–1123.59)	890.90 (710.76–1107.78)	−3.04 (−3.15, −2.93)	−0.84 (−0.9, −0.78)	−3.86 (−3.98, −3.74)
Low-middle SDI	Sudan	682.37 (524.98–882.84)	703.91 (544.23–894.66)	787.13 (604.94–1019.14)	3.16 (3.05, 3.27)	11.82 (11.62, 12.02)	15.35 (15.13, 15.57)
	Morocco	785.99 (599.69–1007.33)	794.75 (609.43–1023.34)	775.37 (592.17–1010.69)	1.11 (1.05, 1.17)	−2.44 (−2.54, −2.34)	−1.35 (−1.42, −1.28)
	Palestine	795.17 (612.41–1025.65)	774.73 (598.11–997.3)	801.31 (616.98–1025.51)	−2.57 (−2.67, −2.47)	3.43 (3.32, 3.54)	0.77 (0.72, 0.82)
	Djibouti	830.731 (626.59–1103.91)	870.74 (658.65–1156.42)	921.92 (695.77–1222.17)	4.82 (4.69, 4.95)	5.88 (5.73, 6.03)	10.98 (10.79, 11.17)
Low SDI	Pakistan	396.82 (319.37–496.95)	402.01 (325.43–502.54)	405.12 (327.72–504.86)	1.31 (1.24, 1.38)	0.77 (0.72, 0.82)	2.09 (2, 2.18)
	Afghanistan	616.71 (472.01–802.38)	634.17 (486.31–822.49)	671.07 (514.99–873.97)	2.83 (2.73, 2.93)	5.82 (5.67, 5.97)	8.81 (8.63, 8.99)
	Yemen	683.96 (532.98–884.38)	702.71 (541.43–911.50)	749.75 (580.76–977.97)	2.74 (2.64, 2.84)	6.69 (6.54, 6.84)	9.62 (9.44, 9.8)
	Somalia	695.87 (521.11–933.68)	703.36 (532.17–932.29)	708.21 (536.02–948.65)	1.08(1.02, 1.14)	0.69(0.64, 0.74)	1.77(1.69, 1.85)

EMR, Eastern Mediterranean Region; SDI, Socio-demographic Index.

In 2019, HF contributed to 249.44 thousand (95% UI: 156.97–363.40) YLDs in EMR; 150.98 thousand (95% UI: 94.36–219.12) YLDs among males and 98.45 thousand (95% UI: 63.21–144.83) YLDs among females. Compared with the global rates, the age-standardised YLD rates of HF increased by 8.79% (95% UI: 8.61%, 8.97%) in the EMR from 58.33 (95% UI: 36.72–85.06) in 1990 to 63.46 (95% UI: 39.82–92.59) in 2019. Despite this increase in the EMR, the age-standardised YLD rates of HF decreased globally by 6.82% (95% UI: −6.98%, −6.66%) from 68.60 (95% UI: 44.34–98.67) to 63.92 (95% UI: 41.49–91.95) between 1990 and 2019 (Table 2 and Fig. 1). The trend was similar in the two other periods examined in this study (1990–2005 and 2005–2019) and also in males and females (Fig. 1 and Supplementary Tables S3 and S4).

**Table 2:** Age-standardised YLD rate (per 100,000 persons) of heart failure in 1990, 2005 and 2019, and their relative percentage change by EMR countries.

		YLD rate			%Δ		
		1990	2005	2019	1990–2005	2005–2019	1990–2019
	Global	68.60 (44.34–98.67)	64.89 (42.30–93.03)	63.92 (41.49–91.95)	−5.41 (−5.55, −5.27)	−1.49 (−1.57, −1.41)	−6.82 (−6.98, −6.66)
	EMR	58.33 (36.72–85.06)	60.91 (38.22–88.77)	63.46 (39.82–92.59)	4.42 (4.29, 4.55)	4.19 (4.07, 4.31)	8.79 (8.61, 8.97)
High SDI	Qatar	78.04 (51.60–110.80)	86.35 (56.68–123.23)	75.48 (48.43–111.75)	10.65 (10.46, 10.84)	−12.59 (−12.8, −12.38)	−3.28 (−3.39, −3.17)
	United Arab Emirates	71.76 (46.08–105.54)	69.51 (44.37–102.59)	78.54 (49.60–116.60)	−3.14 (−3.25, −3.03)	12.99 (12.78, 13.2)	9.45 (9.27, 9.63)
	Kuwait	88.06 (55.23–128.58)	93.13 (58.23–136.39)	90.05 (56.76–131.65)	5.76 (5.62, 5.9)	−3.31 (−3.42, −3.2)	2.26 (2.17, 2.35)
High-middle SDI	Oman	53.29 (34.62–75.38)	66 (41.42–96.08)	69.04 (43.11–99.42)	23.85 (23.59, 24.11)	4.61 (4.48, 4.74)	29.56 (29.28, 29.84)
	Saudi Arabia	57.37 (36.68–85.39)	67.12 (42.60–97.12)	70.23 (43.88–102.43)	16.99 (16.76, 17.22)	4.63 (4.5, 4.76)	22.42 (22.16, 22.68)
	Bahrain	77.78 (49.40–113.18)	70.51 (44.42–101.67)	70.67 (44.70–103.27)	−9.35 (−9.53, −9.17)	0.23 (0.2, 0.26)	−9.14 (−9.32, −8.96)
	Lebanon	74.22 (47.25–108.35)	75.40 (46.85–111.63)	80.47 (51.11–118.47)	1.59 (1.51, 1.67)	6.72 (6.56, 6.88)	8.42 (8.25, 8.59)
	Libya	71.03 (44.22–102.66)	80.01 (50.15–115.95)	80.40 (49.60–117.72)	12.64 (12.43, 12.85)	0.49 (0.45, 0.53)	13.19 (12.98, 13.4)
	Jordan	83.99 (53.44–123.42)	84.73 (54.68–124.66)	86.10 (54.70–126.23)	0.88 (0.82, 0.94)	1.62 (1.54, 1.7)	2.51 (2.41, 2.61)
Middle SDI	Egyspt	64.25 (39.43–95.83)	64.05 (38.37–96.92)	69.04 (42.45–104.32)	−0.31 (−0.34, −0.28)	7.79 (7.62, 7.96)	7.46 (7.3, 7.62)
	Syrian Arab Republic	62.70 (39.25–92.73)	66.59 (41.51–97.40)	69.38 (43.25–103.69)	6.2 (6.05, 6.35)	4.19 (4.07, 4.31)	10.65 (10.46, 10.84)
	Iraq	75.30 (46.76–110.51)	71.17 (44.63–104.51)	72.84 (46.60–108.22)	−5.48 (−5.62, −5.34)	2.35 (2.26, 2.44)	−3.27 (−3.38, −3.16)
	Tunisia	72.20 (44.81–106.54)	73.48 (46.27–107.17)	74.67 (47.63–109.18)	1.77 (1.69, 1.85)	1.62 (1.54, 1.7)	3.42 (3.31, 3.53)
	Iran (Islamic Republic of)	82.92 (53.02–119.88)	80.73 (51.26–116.90)	80.27 (51.22–115.95)	−2.64 (−2.74, −2.54)	−0.57 (−0.62, −0.52)	−3.2 (−3.31, −3.09)
Low-middle SDI	Sudan	60.88 (37.57–89.75)	62.99 (39.68–92.54)	70.69 (44.10–103.36)	3.47 (3.36, 3.58)	12.22 (12.02, 12.42)	16.11 (15.88, 16.34)
	Morocco	70.58 (44.09–104.43)	71.50 (44.67–104.99)	69.96 (43.16–102.03)	1.3 (1.23, 1.37)	−2.15 (−2.24, −2.06)	−0.88 (−0.94, −0.82)
	Palestine	71.29 (44.7–105.11)	69.62 (44.33–101.14)	72.31 (45.64–105.88)	−2.34 (−2.43, −2.25)	3.86 (3.74, 3.98)	1.43 (1.36, 1.5)
	Djibouti	74.06 (45.95–110.76)	77.76 (48.90–114.75)	82.56 (51.67–122.32)	5 (4.86, 5.14)	6.17 (6.02, 6.32)	11.48 (11.28, 11.68)
Low SDI	Pakistan	34.92 (22.34–50.70)	35.37 (22.90–51.14)	35.80 (22.83–52.21)	1.29 (1.22, 1.36)	1.22 (1.15, 1.29)	2.52 (2.42, 2.62)
	Afghanistan	54.87 (34.87–81.64)	56.48 (36.12–82.92)	60 (38.36–88.58)	2.93 (2.83, 3.03)	6.23 (6.08, 6.38)	9.35 (9.17, 9.53)
	Yemen	61.14 (38.62–90.08)	63.05 (39.41–94.43)	67.33 (42.05–99.77)	3.12 (3.01, 3.23)	6.79 (6.63, 6.95)	10.12 (9.93, 10.31)
	Somalia	61.77 (38.08–92.49)	62.45 (38.79–91.23)	63.01 (38.76–94.01)	1.1(1.04, 1.16)	0.9(0.84, 0.96)	2.01(1.92, 2.1)

YLD, years lived with disability; EMR, Eastern Mediterranean Region; SDI, Socio-demographic Index.

### National-level estimates

EMR countries were categorised into five groups based on SDI quintiles as presented in Table 1. There was a considerable variation in age-standardised prevalence rates of HF across the five groups of the EMR (Table 1). Interestingly, the highest age-standardised HF prevalence rate in 2019 was in the high SDI group, namely Kuwait (995.36; 95% UI: 772.49–1279.59) per 100,000, followed by Jordan (951.92; 95% UI: 739.76–1225.12) from the high-middle SDI group, and Djibouti (921.92; 95% UI: 695.77–1222.17) from the low-middle SDI group. Notably, the HF prevalence rate were highest in the high SDI group (ranged from 833.30 to 995.36 per 100,000 persons) followed by the high-middle SDI group with a range of 768.73–951.92 per 100,000 persons and lowest in the low SDI countries (ranged from 405.12 to 749.75 per 100,000 persons). Nevertheless, over the period 1990 to 2019, all countries in the low SDI group had increasing prevalence rates. For example, though Pakistan had the lowest age-standardised HF prevalence rate in all points (396.82, 402.01 and 405.12 per 100,000 persons), this rate had increased over the period shown. Oman showed a noticeable rise by 28.79% (95% UI: 28.51%, 29.07%) in the age-standardised prevalence rate over the period of 30 years, representing the largest change in the EMR, followed by Saudi Arabia (20.97%; 95% UI: 20.72%, 21.22%) and Sudan (15.35%; 95% UI: 15.13%, 15.57%). Although an increase was observed in most EMR countries, the prevalence rates for Bahrain, Iraq, Iran, and Qatar decreased by 9.66% (95% UI: −9.84%, −9.48%), 4.07% (95% UI: −4.19%, −3.95%), 3.86% (95% UI: −3.98%, −3.74%), and 3.78% (95% UI: −3.9%, −3.66%), respectively between 1990 and 2019 (Table 1). For males and females, similar trends of decreased HF prevalence rates were observed in the same period in these four countries (Supplementary Fig. S1). Across the EMR countries, the highest age-standardised prevalence rate was seen among Jordanian males (1196.04; 95% UI: 922.7–1533.31 per 100,000 persons; Supplementary material Table S1) in 2019, while that of females was observed in Kuwait (777.44; 95% UI: 608.33–998.35 per 100,000 persons; Supplementary material Table S2).

The trends of YLDs mirrored those of the prevalence rates (Table 2). In 2019, the EMR high SDI countries had the highest YLD rates with a range of 75.48 (95% UI: 48.43–111.75) to 90.05 (95% UI: 56.76–131.65), followed by high-middle and middle SDI countries with a range of 69.04 (95% UI: 43.11–99.42) to 86.10 (95% UI: 54.70–126.23) per 100,000 persons. The low SDI countries showed the lowest YLD rates, ranged from 35.80 (95% UI: 22.83–52.21) to 67.33 (95% UI: 42.05–99.77) per 100,000 persons. Moreover, during the period 1990 to 2019 among EMR countries, Kuwait had the highest age-standardised YLD rates: 88.06 (95% UI: 55.23–128.58), 93.13 (95% UI: 58.23–136.39), and 90.05 (95% UI: 56.76–131.65) in 1990, 2005, and 2019, respectively, whereas Pakistan had the lowest age-standardised YLD rates (34.92; 95% UI: 22.34–50.70 in 1990, 35.37; 95% UI: 22.90–51.14 in 2005, 35.80; 95% UI: 22.83–52.21 in 2019). Over the period of 30 years, the age-standardised YLDs increased in most EMR countries, considerably for all low-SDI countries. However, the greatest increase was in Oman (29.56%; 95% UI: 29.28%, 29.84%), Saudi Arabia (22.42%; 95% UI: 22.16%, 22.68%), and Sudan (16.11%; 95% UI: 15.88%, 16.34%), while the rates of age-standardised YLDs decreased in Bahrain (−9.14%; 95% UI: −9.32%, −8.96%), Qatar (−3.28%; 95% UI: −3.39%,-3.17%), Iraq (−3.27%; 95% UI: −3.38%,-3.16%), and Iran (−3.20%; 95% UI: −3.31%,-3.09%) (Table 2). The same results were observed for males and females (Supplementary Tables S3 and S4 and Fig. S2). Mirroring the HF prevalence rates, the highest male age-standardised YLDs was in Jordan: 106.76 (95% UI: 66.42–157.86), 105.06 (95% UI: 66.98–156.09) and 108.29 (95% UI: 68.72–158.17) in 1990, 2005 and 2019 respectively (Table S3), while the highest female age-standardised YLDs was in Kuwait: 70 (95% UI: 43.61–103.34), 71.61 (95% UI: 45.48–105.06) and 70.29 (95% UI: 44.11–104.49) (Table S4).

### Contribution of various etiologies

Among all causes of HF, IHD accounted for the highest age-standardised prevalence rate of HF (327.38; 95% UI: 237.76–434.52) in the EMR in 2019, followed by hypertensive heart disease (281.65; 95% UI: 204.52–383.41), rheumatic heart disease (19.73; 95% UI: 14.99–25.84), non-rheumatic valvular heart disease (4.77; 95% UI: 3.13–7.09), and alcoholic cardiomyopathy (1.71; 95% UI: 1.24–2.35) (Table 3). It was the same globally with the difference in non-rheumatic valvular heart disease, which was placed as the third underlying cause of HF. Among most EMR countries, either IHD or hypertensive heart disease ranked first.

**Table 3:** Age-standardised prevalence rate (per 100,000 persons) of heart failure by each underlying cause in 1990 and 2019 by the EMR countries.

		Ischemic heart disease	Hypertensive heart disease	Non-rheumatic valvular heart disease	Rheumatic heart disease	Alcoholic cardiomyopathy
		**1990**				
	Global	306.28 (226.27–395.76)	219.54 (158.77–299.36)	41.71 (27.69–60.31)	24.49 (18.89–31.19)	12.6 (9.6–16.45)
	EMR	298.36 (217.55–397.63)	257.73 (186.8–353.93)	4.55 (2.98–6.85)	20.2 (15.48–26.3)	1.62 (1.17–2.23)
High SDI	Qatar	600.47 (472.43–733.44)	116.6 (89.22–151.98)	17.08 (10.44–26.41)	12.03 (9.41–15.25)	3.58 (2.82–4.49)
	United Arab Emirates	297.09 (213.68–407.67)	403.09 (292.19–547.11)	10.61 (6.71–16.67)	16 (11.83–21.38)	2.08 (1.52–2.88)
	Kuwait	390.47 (284.24–525.22)	512.22 (373.84–682.79)	9.78 (6.08–15.05)	6.37 (4.73–8.3)	1.07 (0.79–1.41)
High-middle SDI	Oman	348.1 (274.56–432.8)	183.72 (141.46–241.6)	7.52 (4.77–12.01)	2.94 (2.23–3.75)	0.92 (0.69–1.2)
	Saudi Arabia	418.04 (303.21–560.83)	87.72 (63.41–119.7)	8.3 (5.23–13.21)	9.47 (6.93–12.51)	1.35 (1–1.81)
	Bahrain	542.63 (400.58–708.62)	158.36 (115.73–218.3)	9.47 (5.94–15)	11.37 (8.45–15.07)	4.84 (3.59–6.42)
	Lebanon	328.09 (237.28–435.69)	410.5 (300.11–556.55)	5.91 (3.82–9)	8.6 (6.32–11.23)	2.82 (2.02–3.86)
	Libya	341.87 (245.24–459.04)	370.77 (272.52–501.18)	6.98 (4.41–10.88)	8.04 (5.93–10.66)	1.91 (1.38–2.69)
	Jordan	327.81 (236.15–438.42)	536.69 (395.1–395.1)	5.36 (3.31–8.42)	5.62 (4.15–7.48)	0.79 (0.58–1.03)
Middle SDI	Egypt	366.22 (247.23–519.21)	270.73 (177.24–399.7)	6.02 (3.93–9.24)	8.85 (5.94–13.06)	2.43 (1.57–3.79)
	Syrian Arab Republic	498.44 (367.54–646.38)	106.13 (76.33–145.15)	11.07 (8.21–15.51)	21.17 (15.58–28.12)	2.02 (1.44–2.8)
	Iraq	480.34 (346.61–630.19)	252.47 (184.74–351.61)	7.55 (4.93–11.53)	15.24 (11.21–20.37)	0.74 (0.57–0.98)
	Tunisia	351.95 (252.56–472.95)	371.76 (267.29–501.57)	5.26 (3.34–8.24)	9.58 (6.99–12.76)	1.72 (1.24–2.3)
	Iran (Islamic Republic of)	408.96 (300.4–540.29)	420.1 (303.05–566.22)	5.11 (3.39–7.42)	17.3 (12.79–23.4)	2.3 (1.67–3.08)
Low-middle SDI	Sudan	285.11 (206.2–389.39)	330.63 (239.59–456.94)	4.34 (2.72–6.8)	12.06 (8.91–16.05)	1.98 (1.39–2.8)
	Morocco	359.64 (261.8–481.35)	353.04 (255.54–483.15)	5.1 (3.24–7.91)	13.71 (10.05–18.21)	2.17 (1.54–3.04)
	Palestine	366.92 (267.11–495.52)	336.88 (245.86–464.75)	4.39 (2.63–7.15)	7.23 (5.29–9.54)	1.13 (0.85–1.48)
	Djibouti	166.23 (106.63–246.05)	299.9 (200.94–428.8)	2.31 (1.49–3.54)	2.4 (1.55–3.64)	1.64 (1.04–2.42)
Low SDI	Pakistan	155.04 (110.86–212.01)	133.49 (92.63–188.53)	1.99 (1.3–2.95)	36.47 (27.36–47.68)	0.68 (0.46–0.96)
	Afghanistan	223.29 (160.66–300.4)	333.31 (243–456.31)	4.32 (2.7–6.82)	14.04 (10.32–18.93)	1.46 (1.03–1.95)
	Yemen	265.64 (189.96–356.38)	351.69 (258.83–479.7)	4.76 (3.02–7.4)	13.59 (9.9–18.32)	1.52 (1.08–2.07)
	Somalia	140.55 (91.16–209.91)	249.59 (167.42–356.78)	1.39 (0.91–2.06)	2.05 (1.29–3.21)	2.77 (1.75–4.13)
		**2019**				
	Global	265.72 (197.23–346.43)	233.77 (170.52–312.9)	31.38 (20.63–46.45)	26.32 (20.4–33.45)	8.51 (6.6–11.01)
	EMR	327.38 (237.76–434.52)	281.65 (204.52–383.41)	4.77 (3.13–7.09)	19.73 (14.99–25.84)	1.71 (1.24–2.35)
High SDI	Qatar	586.91 (429.05–766.08)	105.23 (76.16–143.07)	12.86 (7.92–19.34)	10.08 (7.5–12.86)	3.28 (2.45–4.32)
	United Arab Emirates	339.21 (242.56–455.69)	424.02 (305.19–577.31)	12.88 (8.02–19.69)	16.14 (11.65–21.68)	2.08 (1.48–2.84)
	Kuwait	404.1 (292.57–535.9)	514.94 (376.28–690.61)	8.57 (5.2–12.83)	6.52 (4.87–8.49)	1.08 (0.8–1.43)
High-middle SDI	Oman	475.03 (354.24–619.21)	223.09 (162.42–303.13)	5.66 (3.64–8.5)	3.73 (2.75–4.96)	0.93 (0.67–1.31)
	Saudi Arabia	525.78 (384.19–688.69)	94.86 (69.55–128.96)	8.44 (5.27–12.78)	10.48 (7.71–13.9)	1.52 (1.13–2.05)
	Bahrain	475.64 (348.76–632.62)	145.26 (105.17–197.9)	8.61 (5.4–12.9)	10.62 (7.79–14.1)	4.94 (3.69–6.55)
	Lebanon	370.44 (266.27–502.52)	428.83 (311.97–592.54)	4.44 (2.9–6.59)	9.23 (6.84–12.04)	2.93 (2.13–3.99)
	Libya	416.17 (304.37–555.77)	389.51 (282.69–534.08)	5.94 (3.77–8.99)	8.82 (6.44–11.57)	2.16 (1.55–3.01)
	Jordan	317.68 (230.1–422.54)	561.62 (417.93–747.58)	5.51 (3.33–8.68)	5.84 (4.34–7.63)	0.83 (0.62–1.07)
Middle SDI	Egypt	391.59 (264.34–548.07)	283.14 (183.82–416.49)	6.51 (4.23–9.97)	9.54 (6.42–13.9)	2.79 (1.79–4.35)
	Syrian Arab Republic	557.45 (414.39–719.42)	112.28 (81.12–156.38)	9.59 (7.43–12.63)	22.79 (16.78–30.83)	2.27 (1.62–3.13)
	Iraq	451.57 (322.19–601.55)	249.77 (179.65–349.73)	6.33 (4.17–9.53)	15.54 (11.63–20.33)	0.72 (0.54–0.94)
	Tunisia	364.36 (262.84–484.49)	378.24 (277.52–513.58)	4.84 (3.11–7.35)	9.94 (7.36–13.06)	1.88 (1.39–2.56)
	Iran (Islamic Republic of)	386.97 (286.08–509.43)	406.32 (293.66–546.33)	4.63 (3.08–6.68)	17.53 (12.98–23.82)	2.28 (1.67–3.05)
Low-middle SDI	Sudan	350.4 (249.39–473.78)	364.63 (263.61–499.95)	4.69 (2.99–7.25)	13.75 (10.14–18.17)	2.15 (1.5–2.99)
	Morocco	343.88 (248.64–467.59)	355.93 (258.71–487.66)	5.46 (3.49–8.45)	13.81 (10.27–18.19)	2.26 (1.63–3.09)
	Palestine	371.99 (269.49–496.34)	337 (243.79–466.33)	4.5 (2.67–7.3)	7.22 (5.38–9.57)	1.19 (0.89–1.56)
	Djibouti	198.84 (128.59–292.65)	319.34 (213.56–459.28)	1.91 (1.26–2.92)	2.56 (1.63–3.85)	1.87 (1.17–2.74)
Low SDI	Pakistan	161.09 (114.43–220.05)	138.55 (96.24–196.86)	2.05 (1.34–3.05)	38.43 (28.88–50.4)	0.69 (0.47–0.98)
	Afghanistan	250.64 (180.38–336.6)	357.56 (261.65–491.3)	4.33 (2.7–6.71)	15.25 (11.23–20.23)	1.6 (1.13–2.14)
	Yemen	304.72 (218.29–409.02)	375.92 (274.84–511.56)	4.46 (2.84–6.91)	14.65 (10.86–19.57)	1.6 (1.13–2.22)
	Somalia	149.36 (98.33–217.66)	248.69 (167.52–359.57)	1.13 (0.77–1.7)	2.26 (1.43–3.46)	3.01 (1.92–4.37)

EMR, Eastern Mediterranean Region; SDI, Socio-demographic Index.

IHD had the highest age-standardised YLD rates of HF in the EMR and either IHD or hypertensive heart disease occupied the first rank in most countries of the region (Table 4).

**Table 4:** Age-standardised YLD rate (per 100,000 persons) of heart failure by each underlying cause in 1990 and 2019 by EMR countries.

		Ischemic heart disease	Hypertensive heart disease	Non-rheumatic valvular heart disease	Rheumatic heart disease	Alcoholic cardiomyopathy
		**1990**				
	Global	27.47 (16.85–41.77)	19.66 (11.75–30.51)	3.76 (2.14–6.34)	2.22 (1.38–3.31)	1.15 (0.72–1.7)
	EMR	26.58 (16.14–40.64)	22.98 (13.56–35.67)	0.41 (0.23–0.71)	1.82 (1.14–2.7)	0.15 (0.09–0.23)
High SDI	Qatar	54.02 (35.13–77.77)	10.47 (6.48–15.77)	1.54 (0.8–2.64)	1.1 (0.7–1.68)	0.33 (0.22–0.48)
	United Arab Emirates	26.66 (15.96–40.45)	36.22 (21.95–55.61)	0.96 (0.52–1.69)	1.45 (0.87–2.29)	0.19 (0.12–0.3)
	Kuwait	35.16 (21.43–53.9)	46.08 (27.62–68.58)	0.89 (0.48–1.57)	0.58 (0.37–0.88)	0.1 (0.06–0.15)
High-middle SDI	Oman	31.01 (19.1–44.95)	16.37 (10.05–24.46)	0.67 (0.37–1.19)	0.27 (0.17–0.41)	0.09 (0.05–0.13)
	Saudi Arabia	37.08 (22.97–57.2)	7.79 (4.55–12.08)	0.74 (0.39–1.31)	0.86 (0.53–1.35)	0.12 (0.08–0.19)
	Bahrain	48.71 (30.11–73.2)	14.23 (8.54–22.26)	0.86 (0.46–1.48)	1.04 (0.63–1.56)	0.44 (0.27–0.68)
	Lebanon	29.36 (17.88–44.05)	36.85 (22.07–56.27)	0.54 (0.29–0.93)	0.79 (0.49–1.2)	0.26 (0.16–0.41)
	Libya	30.59 (18.56–46.27)	33.23 (19.95–49.86)	0.63 (0.34–1.11)	0.74 (0.45–1.12)	0.18 (0.11–0.28)
	Jordan	29.42 (17.48–44.81)	48.17 (29.25–73.55)	0.49 (0.26–0.87)	0.52 (0.32–0.79)	0.07 (0.05–0.11)
Middle SDI	Egypt	32.74 (18.76–51.87)	24.21 (13.36–39.46)	0.55 (0.3–0.96)	0.81 (0.47–1.33)	0.23 (0.13–0.38)
	Syrian Arab Republic	44.59 (26.87–67.98)	9.51 (5.58–14.95)	1 (0.6–1.58)	1.92 (1.15–2.98)	0.19 (0.11–0.29)
	Iraq	42.89 (26–65.66)	22.53 (13.39–34.78)	0.68 (0.38–1.17)	1.38 (0.85–2.1)	0.07 (0.04–0.10)
	Tunisia	31.57 (18.81–48.05)	33.42 (19.76–51.48)	0.48 (0.26–0.82)	0.88 (0.54–1.37)	0.16 (0.10–0.25)
	Iran (Islamic Republic of)	36.51 (22.39–56.11)	37.57 (21.97–57.31)	0.46 (0.27–0.76)	1.57 (0.98–2.4)	0.21 (0.13–0.32)
Low-middle SDI	Sudan	25.37 (15.28–38.94)	29.47 (17.45–46.03)	0.39 (0.21–0.69)	1.1 (0.68–1.7)	0.18 (0.11–0.29)
	Morocco	32.25 (19.37–49.15)	31.66 (18.91–49.14)	0.46 (0.25–0.81)	1.25 (0.77–1.94)	0.20 (0.12–0.31)
	Palestine	32.85 (20.12–49.83)	30.12 (17.85–46.99)	0.4 (0.2–0.73)	0.67 (0.41–1.02)	0.1 (0.07–0.16)
	Djibouti	14.84 (8.29–24.57)	26.75 (14.97–42.33)	0.21 (0.12–0.37)	0.22 (0.12–0.37)	0.15 (0.09–0.25)
Low SDI	Pakistan	13.6 (8.23–20.74)	11.7 (6.71–18.52)	0.18 (0.1–0.31)	3.26 (1.98–4.82)	0.06 (0.04–0.10)
	Afghanistan	19.82 (11.83–30.36)	29.61 (17.95–45.91)	0.39 (0.21–0.7)	1.27 (0.77–1.97)	0.13 (0.08–0.21)
	Yemen	23.71 (14.16–36.76)	31.38 (18.85–47.92)	0.43 (0.23–0.74)	1.23 (0.75–1.91)	0.14 (0.08–0.22)
	Somalia	12.49 (6.94–20.71)	22.18 (12.37–35.47)	0.13 (0.07–0.22)	0.19 (0.1–0.32)	0.25 (0.14–0.42)
		**2019**				
	Global	23.86 (14.74–35.69)	21.01 (12.61–32.68)	2.83 (1.59–4.73)	2.39 (1.50–3.57)	0.78 (0.49–1.16)
	EMR	29.37 (17.76–44.96)	25.28 (14.86–39.27)	0.44 (0.24–0.75)	1.79 (1.1–2.66)	0.16 (0.10–0.24)
High SDI	Qatar	53.08 (33–80.88)	9.55 (5.69–14.63)	1.17 (0.62–2.01)	0.92 (0.57–1.39)	0.30 (0.19–0.46)
	United Arab Emirates	30.6 (18.27–46.52)	38.34 (22.92–59.27)	1.17 (0.62–2.05)	1.47 (0.87–2.30)	0.19 (0.12–0.30)
	Kuwait	36.54 (21.94–55.27)	46.54 (28.14–70.72)	0.78 (0.42–1.34)	0.6 (0.38–0.90)	0.10 (0.06–0.15)
High-middle SDI	Oman	42.63 (26.14–63.18)	19.99 (12.03–31.05)	0.51 (0.28–0.89)	0.35 (0.21–0.51)	0.09 (0.05–0.13)
	Saudi Arabia	47.25 (28.32–72.07)	8.54 (5.03–13.36)	0.77 (0.42–1.37)	0.96 (0.60–1.48)	0.14 (0.09–0.21)
	Bahrain	42.95 (26.39–64.13)	13.11 (7.94–20.13)	0.79 (0.43–1.37)	0.97 (0.59–1.48)	0.46 (0.28–0.69)
	Lebanon	33.38 (20.2–51.38)	38.71 (23.28–59.52)	0.41 (0.22–0.71)	0.85 (0.53–1.29)	0.27 (0.17–0.42)
	Libya	37.42 (22.34–58.03)	35.08 (21.10–54.43)	0.54 (0.29–0.96)	0.81 (0.50–1.23)	0.20 (0.12–0.32)
	Jordan	28.7 (17.1–43.91)	50.76 (30.38–77.26)	0.5 (0.27–0.91)	0.54 (0.34–0.82)	0.08 (0.05–0.12)
Middle SDI	Egypt	35.2 (20.41–56.82)	25.52 (13.90–42.37)	0.6 (0.33–1.04)	0.87 (0.50–1.46)	0.26 (0.14–0.43)
	Syrian Arab Republic	50.19 (30.52–74.8)	10.11 (6.05–15.78)	0.87 (0.53–1.37)	2.08 (1.26–3.24)	0.21 (0.13–0.33)
	Iraq	40.67 (24.15–62.94)	22.49 (13.2–35.04)	0.58 (0.32–0.97)	1.42 (0.83–2.15)	0.07 (0.04–0.10)
	Tunisia	32.88 (19.71–50.45)	34.18 (20.69–52.82)	0.44 (0.24–0.77)	0.91 (0.57–1.37)	0.17 (0.11–0.27)
	Iran (Islamic Republic of)	34.8 (21.44–53.63)	36.58 (21.61–55.76)	0.42 (0.24–0.69)	1.60 (1–2.50)	0.21 (0.13–0.32)
Low-middle SDI	Sudan	31.42 (18.87–48.52)	32.71 (19.27–50.25)	0.43 (0.23–0.75)	1.26 (0.75–1.91)	0.20 (0.12–0.31)
	Morocco	30.97 (18.54–47.75)	32.1 (18.84–49.42)	0.5 (0.27–0.86)	1.26 (0.76–1.93)	0.21 (0.13–0.33)
	Palestine	33.54 (20.06–51.06)	30.34 (18.07–46.36)	0.41 (0.21–0.74)	0.67 (0.41–1.02)	0.11 (0.07–0.17)
	Djibouti	17.8 (9.82–29.63)	28.64 (15.73–45.17)	0.18 (0.10–0.31)	0.24 (0.13–0.40)	0.17 (0.10–0.28)
Low SDI	Pakistan	14.2 (8.34–22.02)	12.2 (6.96–19.67)	0.19 (0.11–0.32)	3.45 (2.09–5.15)	0.06 (0.04–0.10)
	Afghanistan	22.35 (13.47–34.14)	31.94 (19.28–49.49)	0.39 (0.21–0.69)	1.39 (0.84–2.15)	0.15 (0.09–0.23)
	Yemen	27.3 (16.43–42.52)	33.75 (20.02–51.07)	0.41 (0.22–0.71)	1.34 (0.83–2)	0.15 (0.09–0.23)
	Somalia	13.29 (7.42–21.81)	22.15 (11.85–35.62)	0.1 (0.06–0.17)	0.21 (0.11–0.35)	0.28 (0.15–0.44)

YLD, years lived with disability; EMR, Eastern Mediterranean Region; SDI, Socio-demographic Index.

### Age and sex patterns

**Fig. 2:**
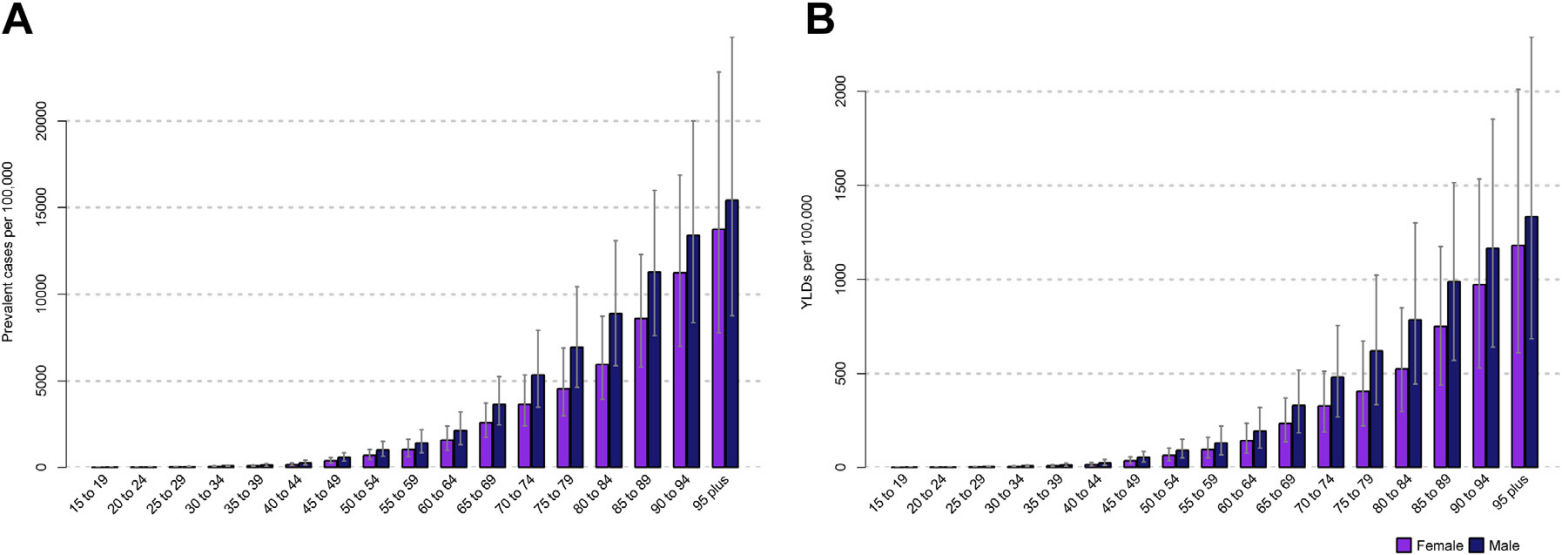
**A- Age-specific prevalence rate, and B- Age-specific YLD rate of heart failure (per 100,000 persons) for males and females in the EMR, 2019**. Error bars indicate 95% uncertainty intervals. YLD, years lived with disability; EMR, Eastern Mediterranean Region.

By age group, the prevalence and YLD rates of HF increased with age in both sexes in the EMR in 2019. Males had relatively higher rates than females in all age groups (Fig. 2).

Comparing males to females, in terms of the age-standardised prevalence rate of HF due to each cause, the ranking for IHD was relatively higher in males than females, whereas the ranking for hypertensive heart disease was relatively higher in females than males. The result was the same for most countries in the EMR (Supplementary Tables S5 and.S6).

Evaluating age-standardised YLD rates of HF due to each cause, IHD and hypertensive heart disease ranked first among males and females (Supplementary Tables S7 and S8).

**Fig. 3:**
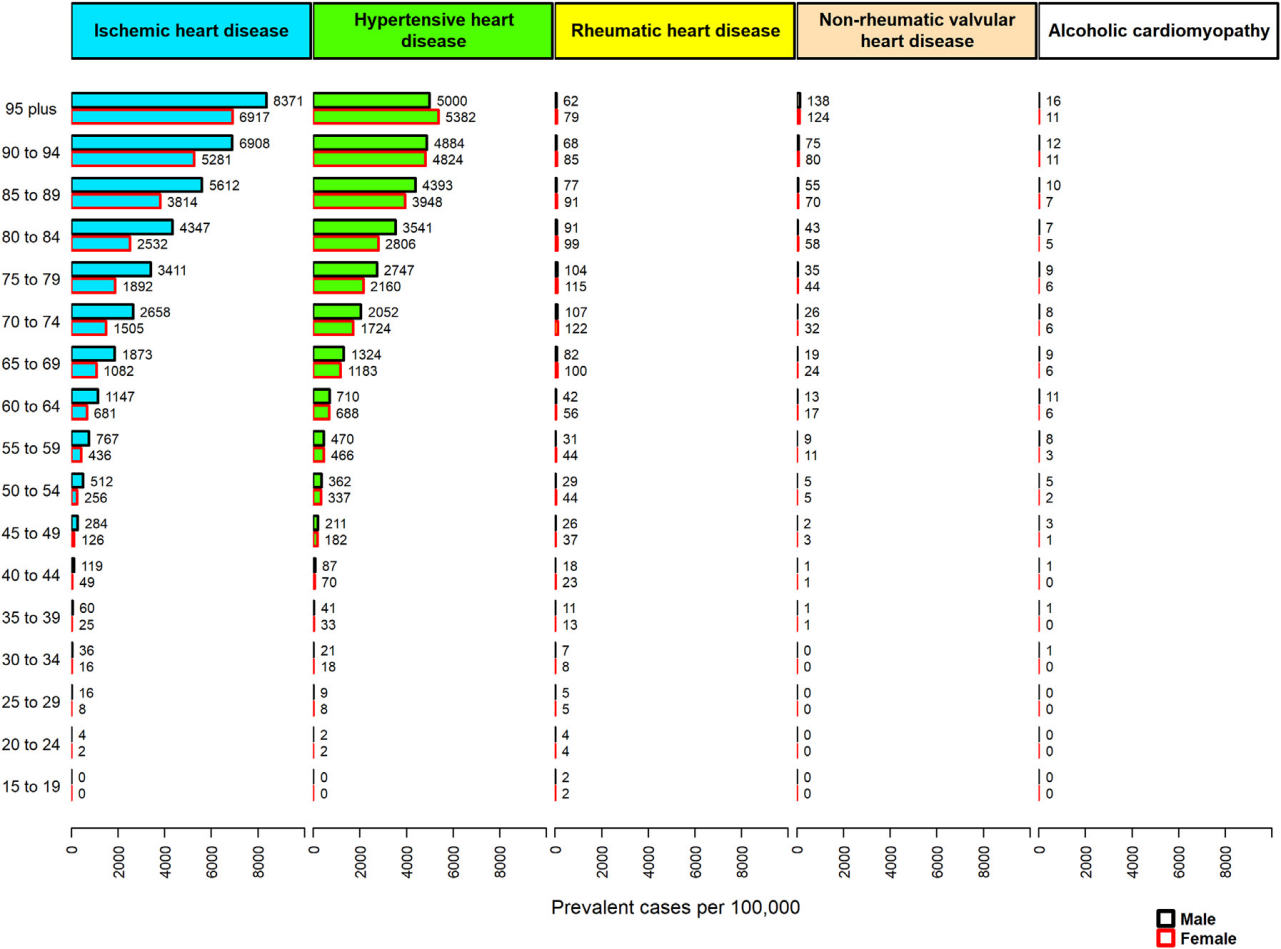
**The prevalence rates of heart failure by each cause and age group in the EMR, 2019.** EMR, Eastern Mediterranean Region.

**Fig. 4:**
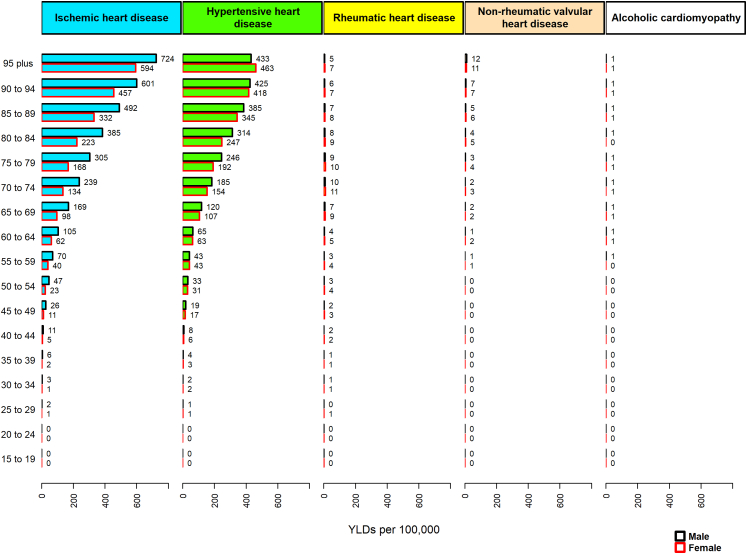
**The YLD rates of heart failure by each cause and age group in the EMR, 2019**. YLD, years lived with disability; EMR, Eastern Mediterranean Region.s.

In adult males ≥ 45 years old, IHD was the main underlying cause among all causes of HF. While in adult females ≥ 35 years old, hypertensive heart disease was the primary underlying cause of HF. In the elderly ≥ 85 years old, IHD was the main underlying cause of HF in both sexes. The alcoholic cardiomyopathy effect on HF was mainly concentrated in males of all ages (Figs. 3 and 4).

### Burden of HF by SDI and HAQ index

Supplementary Fig. S3 depicted the relationship between HAQ Index and SDI between 1990 and 2016. Overall, it can see that the highest observed HAQ Index values increased by a rise in SDI. All countries with various SDI significantly increased their HAQ Index score. Kuwait and Qatar showed the largest improvements among high-SDI countries, and Lebanon, Oman, and Saudi Arabia had the most progress among high-middle-SDI countries. For low middle-SDI and low-SDI countries (for example Djibouti, Pakistan, Afghanistan, Somalia), advances in the HAQ Index either primarily took place or accelerated from 2000 to 2016.

Further evaluation was done by considering the burden of HF based on SDI and HAQ Index levels. Supplementary Figs. S4 and S5 illustrated the relationship between age-standardised prevalence and YLD rates and SDI for each country-year from 1990 to 2019. The majority of countries showed positive change in SDI even though those countries in the higher quintile of SDI indicated a great increase in the burden of HF (Oman, Saudi Arabia, United Arab Emirates) and others in lower quintiles have not considerably changed or saw only slight growth in HF burden, over the period shown. The countries that achieved high SDI quintiles (Kuwait and Qatar) increased to their peak point of HF burden after a decade before dramatic decline over the next 10 years. Burden of HF in Bahrain as a high-middle country dwindled over 30 years. Precisely, a similar pattern was observed by assessing the relationship between age-standardised prevalence and YLD rates and HAQ Index for each country-year from 1990 to 2019 (Supplementary Figs. S6 and S7).

## Discussion

Our results indicate that the burden of HF remains dramatically high in the EMR, as a whole, and in most of the countries of the region. There was an increase in the age-standardised prevalence and YLD rates, while the global rates of these two measures have decreased during 1990–2019. There was also no substantial difference in the etiology spectrum of HF across the 22 countries. Overall, IHD and hypertensive heart disease were the most common underlying causes of HF in the EMR. IHD was the major underlying cause among males, and hypertensive heart disease among females. However, the effects of these two underlying causes were mainly concentrated in adults. Other studies also showed that the underlying causes of HF varied widely across regions, mainly because of different risk factor exposure.^17^ HF was considered as a developing pandemic, with an extensive epidemiological difference and wide variety of heterogeneity reasons among regions and countries.^4^^,^^6^ It is a highly complex clinical syndrome involving many factors, such as sociodemographic status, resource accessibility, implementation of adequate preventive strategies, and effective clinical interventions. Thus, awareness of HF burden in EMR informs the health decision makers in EMR countries so that they should pay a particular attention to the association between equity and burden of HF and opt for cost effective strategies based on early diagnosis and prevention.^4^^,^^18^

EMR witnessed drastic and considerable changes during several decades with respect to risk factors, sociodemographic profiles, health indicators, health system capacities and coverage, and war, conflict and social or political unrest.

Some Gulf Cooperation Council (GCC) countries and also high-middle and middle SDI countries such as Jordan, Lebanon, Iran, and Tunisia have devised their own operational policy and course of action for risk factors. Nevertheless, all low SDI countries and many others in this region drastically suffer from lacking the necessary policy and strategy to encounter the ever-escalating risk factors at national level. As a result, the more a country is exposed to risk factors, the more it will be affected by the burden of HF.^19^, ^20^, ^21^, ^22^

Having socioeconomic status (SES) terms in mind, it is proven that countries with a high level of SDI quintile categories have managed to reduce the burden of HF considerably. This is the direct result of a better healthcare system with superior treatment programs in these countries.^23^ Furthermore, countries with low or low-middle SDI quintiles, such as Sudan, Yemen, and Djibouti have a growing rate of HF prevalence that is in itself pertinent to the high underlying cause and risk factors, including DM, HTN, and unhealthy lifestyles such as smoking, obesity, sedentary lifestyle, unhealthy diet in the previously mentioned areas. Nevertheless, in this region, countries with various socioeconomic levels and SDI quintiles are still far behind most other WHO regions. Poor compliance and unavailability or unaffordability of the most important CVD medicines were reported in low- and middle-SDI countries, as well as underutilisation of required care and services in high SDI countries.^24^, ^25^, ^26^

An additional component is the weak health system with limited capacity for screening, early diagnosis, and treatment.^13^ Since effectual health care of individuals would consequently increase health outcomes and decrease mortality, having access to first-rate and excellent health care is the ultimate aim of health systems. However, as it is suspected, providing a systematic and well-planned healthcare system that would benefit all without imposing huge and hefty financial burden on people is quite challenging. The inadequacy of integrating services into primary health care and substandard quality of health care is also quite problematic. Moreover, health care services' safety and security in conflict zones are also of major significance.^27^ In this regard, the Prospective Urban Rural Epidemiology (PURE) study showed marked variation in percentage access and use of required care and services, availability, affordability, and medications for CVD between and within countries.^26^ Therefore, factors such as accessibility, affordability, acceptability, and quality of medicines and care, as well as compliance to medical recommendations and drug adherence are important issues in EMR and might be responsible for the high burden of HF in the region.^22^^,^^28^, ^29^, ^30^ What is more, the diagnosis of HF is more likely subject to selection biases due to screening and health service provision than acute outcomes such as myocardial infarction. As the analysis of SDI and HAQ showed, the countries variations in HF might be due to factors like healthcare quality, access, and infrastructure. Their effects can interact in a way that influence both incidence and diagnosis rate. In spite of the fact that potential reduction in HF incidence can be the result of early diagnosis and treatment of underlying causes of HF (e.g., HTN), over short-term better access to healthcare could also increase diagnosis rate.^3^ In conclusion, if any strategies and health care plans are to yield any benefit in middle-to-low SDI countries, they should be conducted at maximum speed. Thus, national and international agencies should muster all their efforts to reinforce the already existing and deteriorating health system so that everyone, even the poorest, can access to high-quality treatment.

When basic health indicators have been investigated in various countries such as those inflicted by war and other various types of turmoil, the gross discrepancy has been indicated. More importantly, if the wars and unrest were accompanied by aging and population growth, the region would face extreme difficulty in its health system. In spite of the fact that a study reported that from 1990 to 2010, some countries in EMR (such as Egypt, Libya, Syria, and Yemen) had a steady increase in life expectancy, they have lost life expectancy due to the uprising that began in 2010. Therefore, even though some countries in the region have historically seen improvements in life expectancy and other health indicators, the current situation will definitely damage the health conditions and have a negative effect on the region. Based on the extensive research that has been conducted and its findings, it is suggested that the countries endeavour to reduce the conflicts and invest more than ever on their health systems.^31^ In addition, the aforementioned conflicts will cause serious harm to the social and health system of the region, and they will accordingly lead to unstable economics, gender and health inequalities. Having in mind the ever-enlarging conflicts of the region since 2010, we can understand the increasing difficulties and inequalities caused as a direct and indirect impact of them.^32^ Therefore, differences in HF can be explained by several aspects of inequities.

One of the factors resulting in social inequities, such as the unjust and avoidable incongruencies in health status within and between countries, is social determinants.^33^ Many researches show that health inequality within a country directly results from socioeconomic inequities. Wealth and income inequity are the most important factors which play a significant negative role in a country that is deteriorating more than other countries or regions in its effort to bring equity to its economic and social system. It is also important to note that EMR has the highest rate of gender inequality in comparison to other regions. It has been stated by the World Bank that women with better educations are most likely to be hired and thus have higher incomes. Besides, the more educated women are, the more knowledge they possess about health care and nutrition.^32^ Numerous studies also showed that compared to men, women with HF were older, less educated, had less income, and received less defibrillator or cardiac resynchronization therapy (CRT) as well as less follow-up care with cardiology specialists. Although the population of EMR, for the most part, consists of the young, but it is predicted that the population of the old will significantly increase in the next 50 years. In the EMR countries, there is a paucity of policies concerning the aging group of the population. As so, all the countries in the region should try to create the opportunity and the proper environment where the elderly can lead a healthy, physically and socially active life. There are various approaches that can a region achieve this environment such as through appropriate public health, prevention interventions, and support for physical and cognitive activities.^18^^,^^32^ All mentioned factors raise the equity problem between and within countries which should be considered.

It has also been proven that the economic sanctions imposed upon some EMR countries also has a direct and indirect bleak impact on the public health system of these countries. But it should be noted that economic sanctions are not the only source of hardship that can influence a country's health system. Some of the many impacts of economic sanctions are those of access to drugs and inflation leading to currency devaluation. As a result, sanctioned countries will face lack of resources and medical supplies, shortage of equipment, etc. Besides, sanctions can damage social determinants of health and, as a result, lead to unemployment and expanding social inequities.^34^^,^^35^

In a more recent case, we have witnessed how the Coronavirus disease (COVID-19) pandemic unveiled the so far hidden and underlying inequities that exist in the social and health system of the countries. Being forced to work in unsanitary environments and crowded places has been a major cause of infection and mortality during the pandemic. However, as the pandemic started in lately 2019 and we reported 2019 GBD data, it could not have a significant effect on reported findings. In addition, WHO reported some other major outbreaks of infectious diseases in some other countries of the region.^36^ A systematic review also briefly described several infectious disease outbreaks that have occurred in the EMR and that are perpetuated by deteriorating healthcare systems associated with local and regional conflicts.^37^ Many countries are severely under the influence of humanitarian emergencies and a great number of refugees, which lead to the decline of the health systems. Other risk factors involving the outbreak of infectious diseases are rapid or unplanned urbanisation, climate change, increased human–animal interaction, weak surveillance and limited laboratory diagnostic capacity, poor infection prevention and control measures in health facilities, and insufficient risk communication and community engagement efforts. Many countries, despite their effort, have faced difficulties in their endeavour for a rapid response in case of an infectious disease outbreak. In such cases, these countries have encountered acute emergencies which their inferior health system fails to respond properly to them. Many countries in the region lack the essential and necessary investment to improve their low-quality health system and thus become more prepared in case of emergencies and outbreaks.^36^^,^^37^ It should be noted that infections, in the majority of instances, cause hospitalisation due to heart failure with reduced ejection fraction (HFrEF) and they are thus followed by a high mortality rate. If a person is inflicted by repeated instances of infection, rehospitalisation becomes a major burden to the country and its health system. In conclusion, strategies and policies should be devised in order to prevent, identify and treat infections. These policies to improve the public health and social environment can help the countries in the region to help people with HFrEF.^38^

As a result, complicated components such as the high prevalence of risk factors, social, political, economic and gender inequalities, social and political conflicts and war still pose high threats to the health and safety of EMR countries. The region needs extensive comprehensive approaches to enable countries to enhance their endeavours so that they should take urgent action to improve social determinants and access and quality of care, move towards global peace, reduce inequality and boost economic growth. The progress made toward achieving the targets has a positive effect on the burden of HF as a major public health problem in the region.

This study has a number of limitations, in spite of its strength, as the first and most comprehensive analysis of the burden of HF and underlying causes in 22 EMR countries. First, it was subject to all the limitations experienced by all other GBD studies. The most important one is the heterogeneity of data collection methods and sources and the quality and completeness of data, which might be related to socioeconomic groupings and health system development. In some cases, an apparent low incidence/prevalence of HF might be due to inadequate reporting and diagnostic capacity. Particularly in the countries, that suffered wars and migration in the past decade, where any form of statistics would be not available and accurate and countries with no registry system such as electronic hospital or health system and medical records. Second, there was a lack of accurate data on HF in some countries in the region. The standard GBD methodology used covariates associated with HF and/or trends in neighboring countries to estimate the epidemiologic measures for these countries. There is a significant variation among these neighboring countries that makes no sense to compare them with each other. Third, data on important measures, including HF incidence and mortality, were missing. Finally, data on the various phenotypes of patients with HF was absent such as HF with reduced or preserved ejection fraction (HFrEF or HFpEF). Another important limitation is that approximately half of the patients with signs and symptoms of HF have a left ventricular ejection fraction (LVEF) that is not markedly decreased. Nevertheless, in our study, due to limited sources in the EMR region, we could not provide a systematic analysis of the burden of HFpEF.

In conclusion, this study provides information on the burden of HF in the EMR and its countries. The study findings will guide policymakers to monitor the situation and strengthen efforts to designing and conducting comprehensive strategies for preventing and controlling the underlying causes, and improving the medical care of HF to reduce its future burden in the region. In addition, these findings could be useful in the implementation of guidelines in the areas of medication and medical service accessibility, clinical practice patterns, and geographically specific public health policy, as well as tackling other hazards threatening the region such as inequality (social, political, economic, and gender), and social and political conflicts, and war. However, some important issues should be taken into account. In spite of the fact that the burden of HF is rising in some EMR countries over the period shown, because of the slight improvement in data collection or reporting thanks to general advance in technology, however, we can see a lower prevalence in these countries which may be related to a significant under reporting. Therefore, it likely does not reflect the actual numbers and ongoing monitoring and analysis of HF burden trends using reliable data would be crucial for public health strategic planning.

## Contributors

All analysis presented in this manuscript were based on data extracted from Institute of Health Metrics and Evaluation (IHME) provided by the GBD core team. RH, KMZ, ML, JAK, SS, AHM, and NS worked on the data and provided critical feedback on the analysis. RH, and KMZ designed and developed the figures and tables. RH, DS, KIIA, AMH, KMZ, ML, JAK, HFAR, SS, IF, WAA, SMSI, AHM and NS provided feedback on methods, results and discussion parts of all manuscript revised versions. RH and DS wrote the first draft of the manuscript. AHM and NS revised the manuscript critically for important intellectual content. RH, AHM, and NS contributed to managing the overall research enterprise. RH and NS managed publications process, had full access to all the data in the study, and had final responsibility for the decision to submit for publication. All authors read and approved the final version.

## Data sharing statement

All GBD 2019 data are publicly available and can be downloaded via the Global Burden of Disease Results Tool (http://ghdx.healthdata.org/gbdresults-tool).

## Declaration of interests

SMSI declares grants from the National Health and Medical Research Council, and the National Heart Foundation. All other authors declare no competing interests.

## Appendix A Supplementary data

Supplementary Figs. S1–S7 and Tables S1–S8

Caption for supplementary material
