# Supplementary material for: Changes in the burden and underlying causes of heart failure in the Eastern Mediterranean Region, 1990–2019: An analysis of the Global Burden of Disease Study 2019
Source: eClinicalMedicine. 2022 Dec 26;56:101788. doi: 10.1016/j.eclinm.2022.101788 (PMC9803705; doi:10.1016/j.eclinm.2022.101788)
Supplement: Supplementary Figs. S1–S7 and Tables S1–S8 [file mmc1.pdf]

Supplementary material

Data sources

A large number of data sources was used by GBD 2019 such as surveys, epidemiological studies, systematic review and meta-analysis, censuses, vital statistics, and other health and population-related data sources including, Population Health Research Institute, World Health Organization Regional Office for the Eastern Mediterranean (EMRO-WHO), Ministry of Health, Central Statistical Organization, General Secretariat for Development Planning, Ministry of Development Planning and Statistics, National Health Authority, International Statistical Institute, Population Council (Interim Demographic and Health Survey), Central Organization for Statistics and Information Technology, General Information Authority, Committee for Health and Social Insurance, Public Commission for Health Care Planning, National Institute of Public Health, Comprehensive Health and Education Forum International.

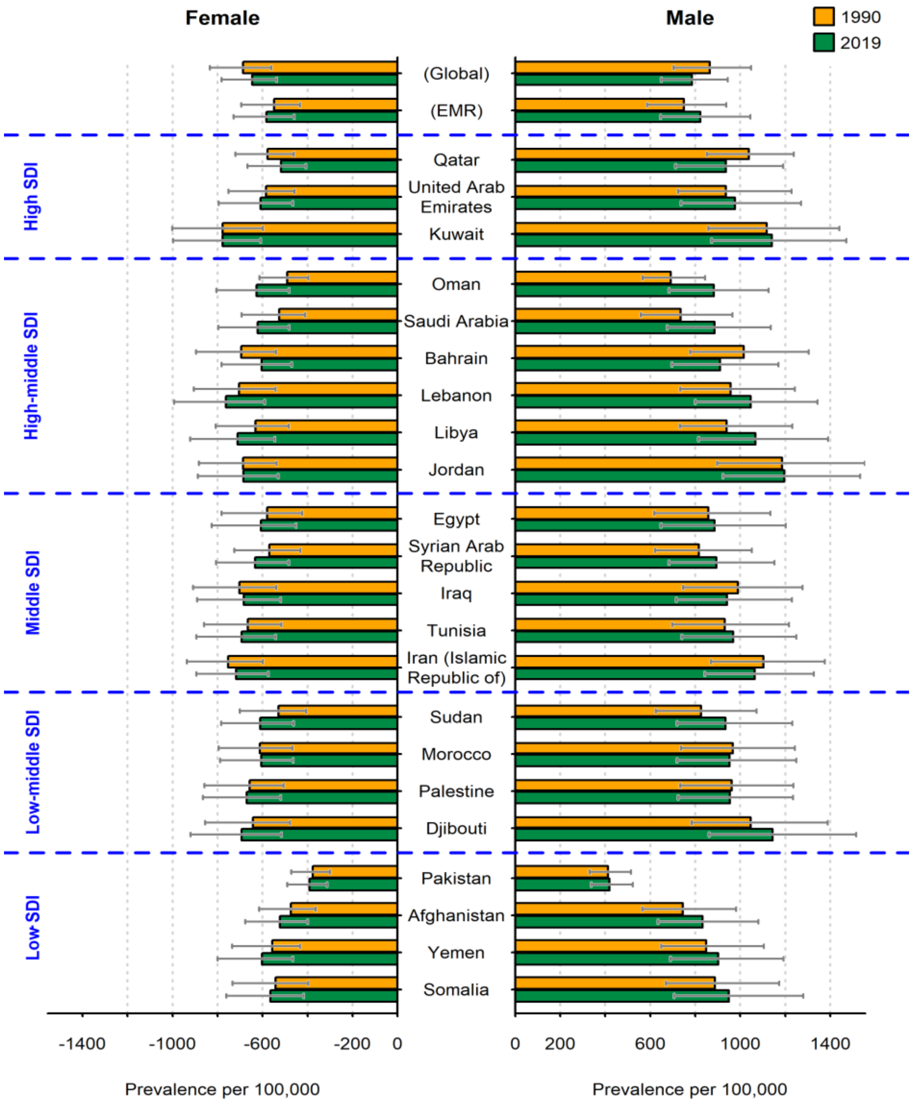

Figure S1: Age-standardised prevalence rate of heart failure (per 100,000 persons) for males and females in EMR countries in 1990 and 2019.

Error bars indicate 95% uncertainty intervals. EMR=Eastern Mediterranean Region. SDI=Socio-demographic Index

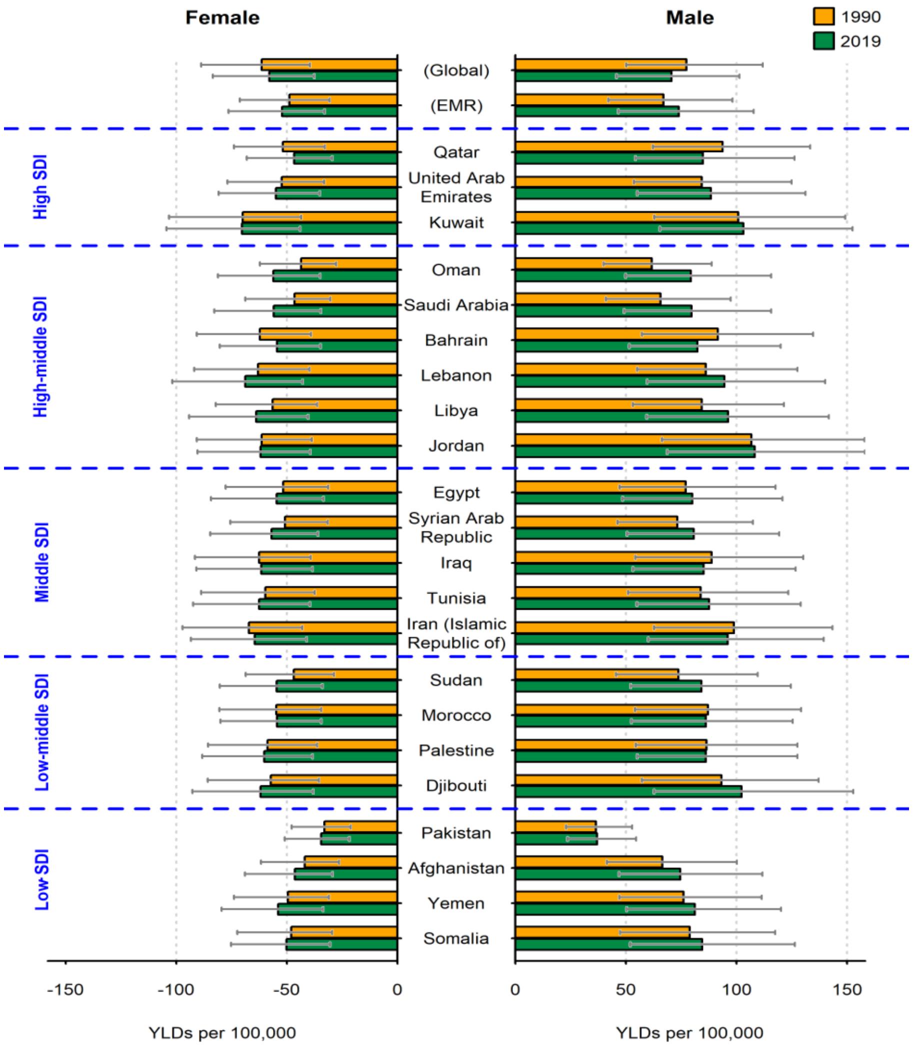

Figure S2: Age-standardised YLD rate of heart failure (per 100,000 persons) for males and females in EMR countries in 1990 and 2019.

Error bars indicate 95% uncertainty intervals. YLD= years lived with disability. EMR=Eastern Mediterranean Region. SDI=Socio-demographic Index

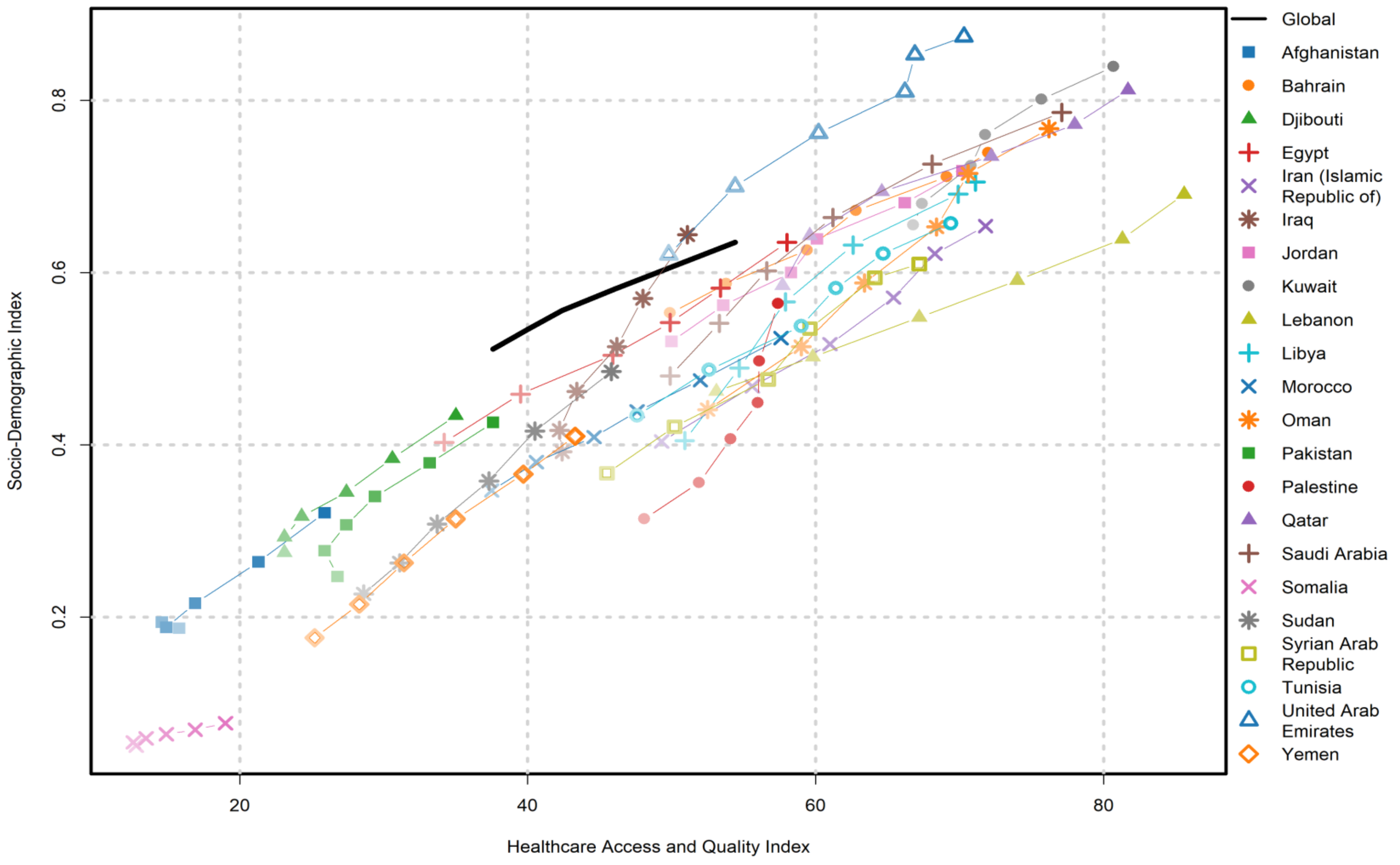

Figure S3: The HAQ Index and level of SDI for 22 EMR countries in 1990,1995, 2000,2005, 2010 and 2016.

For each country, points from left to right illustrate estimates for each 5-year from 1990 and 2016.

HAQ= Healthcare Access and Quality. SDI=Socio-demographic Index. EMR=Eastern Mediterranean Region.

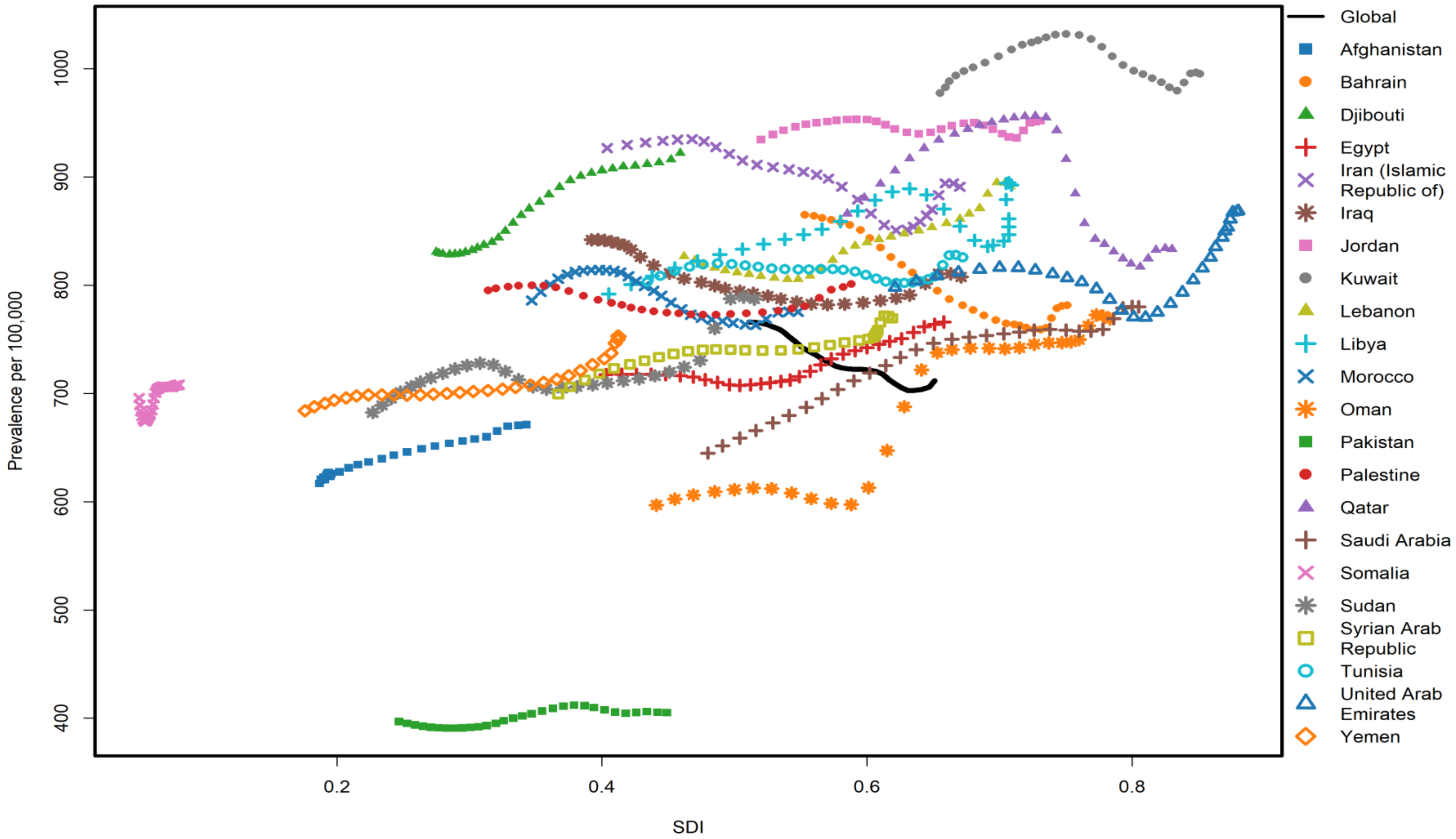

Figure S4: Trends in age-standardised prevalence rate of heart failure for 22 EMR countries by SDI, 1990–2019.

For each country, points from left to right illustrate estimates for each year from 1990 to 2019.

SDI=Socio-demographic Index. EMR=Eastern Mediterranean Region.

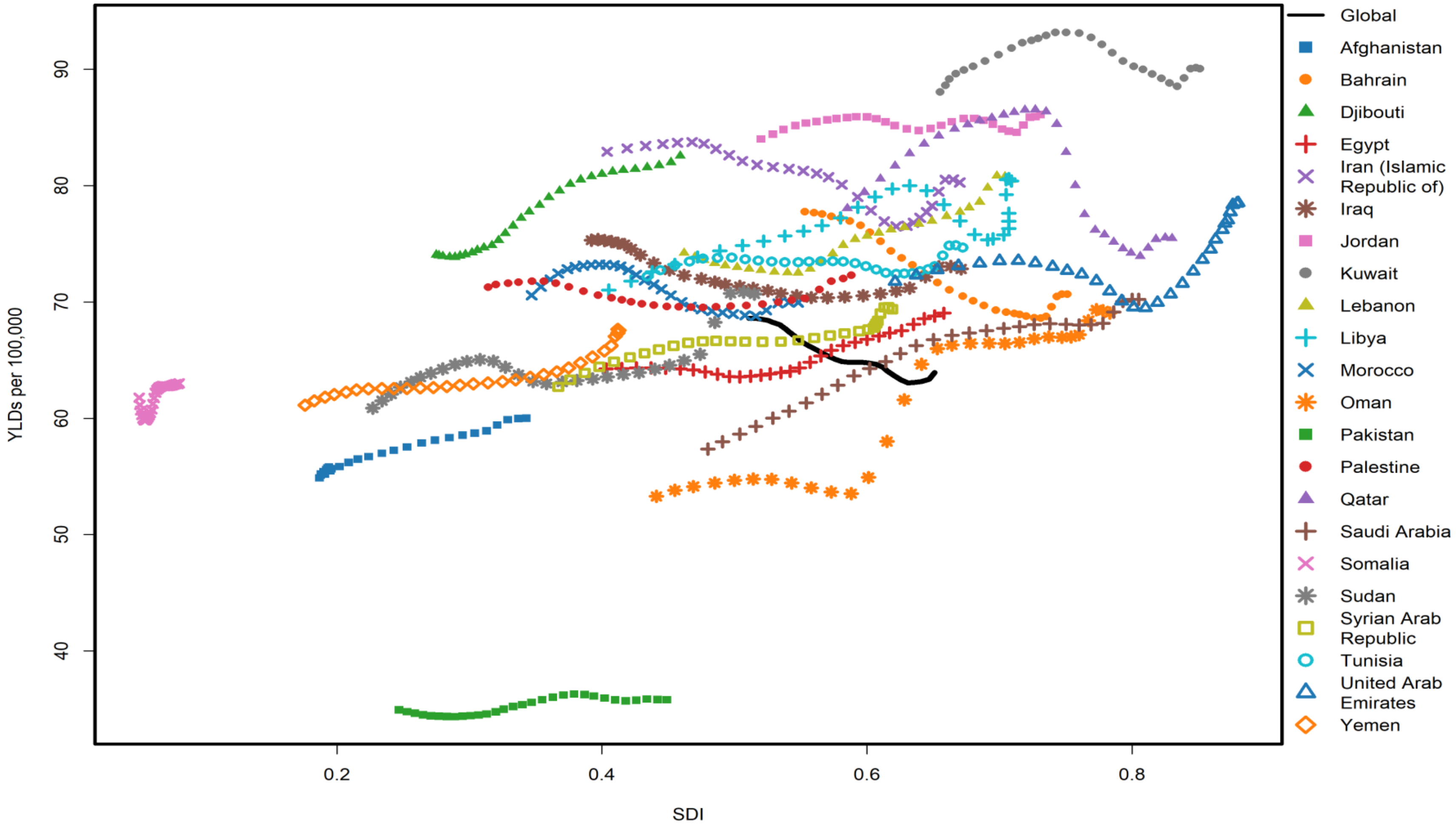

Figure S5: Trends in age-standardised YLD rate of heart failure for 22 EMR countries by SDI, 1990–2019.

For each country, points from left to right illustrate estimates for each year from 1990 to 2019.

YLD= years lived with disability. SDI=Socio-demographic Index. EMR=Eastern Mediterranean Region.

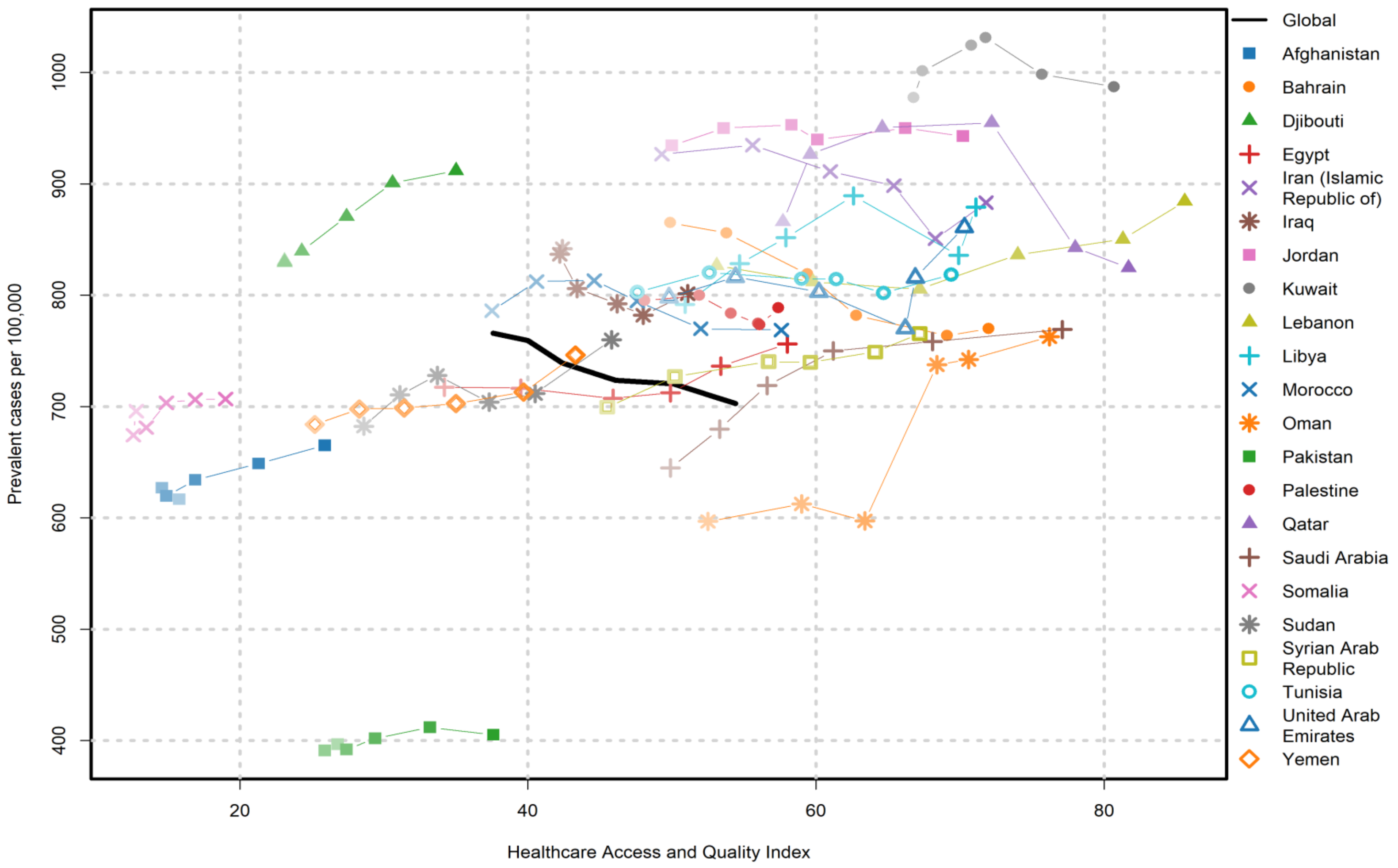

Figure S6: Trends in age-standardised prevalence rate of heart failure for 22 EMR countries by HAQ Index in 1990,1995, 2000,2005,2010 and 2016.

For each country, points from left to right illustrate estimates for each 5-year from 1990 and 2016.

HAQ= Healthcare Access and Quality. EMR=Eastern Mediterranean Region.

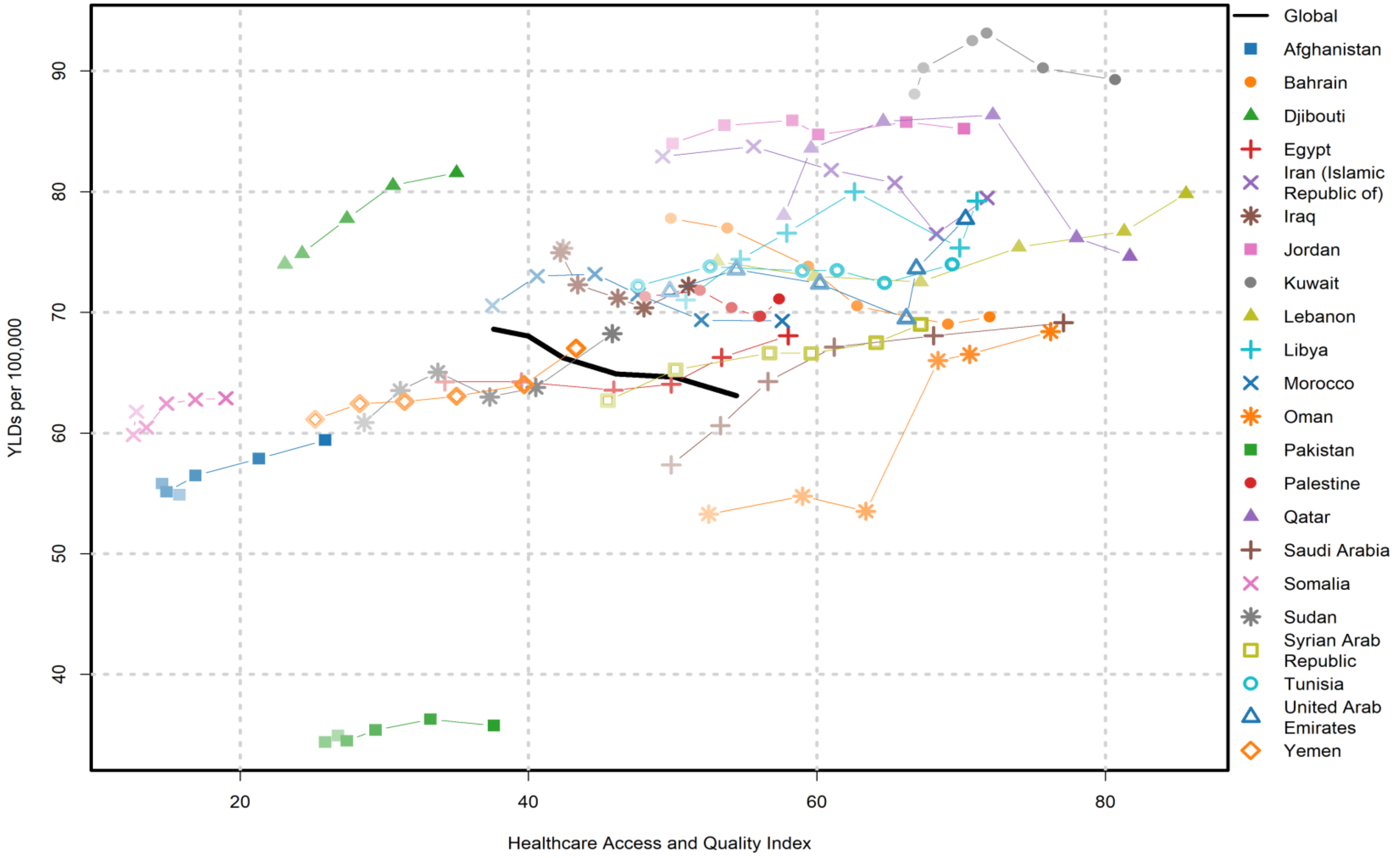

Figure S7: Trends in age-standardised YLD rate of heart failure for 22 EMR countries by HAQ Index in 1990,1995, 2000,2005,2010 and 2016.

For each country, points from left to right illustrate estimates for each 5-year from 1990 and 2016.

YLD= years lived with disability. HAQ= Healthcare Access and Quality. EMR=Eastern Mediterranean Region.

Table S1: Age-standardised prevalence rate (per 100,000 persons) of heart failure for males in 1990,2005 and 2019, and their relative percentage change by EMR countries.

	Prevalence rate			%∆		
	**1990**	**2005**	**2019**	**1990-2005**	**2005-2019**	**1990-2019**
Global	864.25 (703.71-1048.41)	805.81 (669.21-969.47)	785.71 (649.52-944.9)	-6.76(-6.92,-6.6)	-2.49(-2.59,-2.39)	-9.09(-9.27,-8.91)
EMR	749.25 (586.25-938.21)	786.25 (619.51-992.01)	822.27 (646.51-1044.66)	4.94(4.81,5.07)	4.58(4.45,4.71)	9.75(9.57,9.93)
Afghanistan	745.51 (564.71-981.83)	775.05 (589.89-1033.18)	832.74 (634.13-1081.14)	3.96(3.84,4.08)	7.44(7.28,7.6)	11.7(11.5,11.9)
Bahrain	1016.54 (776.89-1303.93)	920.59 (699.65-1188.91)	910.2 (696.5-1170.3)	-9.44(-9.62,-9.26)	-1.13(-1.2,-1.06)	-10.46(-10.65,-10.27)
Djibouti	1046.49 (784.08-1388.42)	1087.65 (817.2-1449.46)	1144.03 (861.69-1515.68)	3.93(3.81,4.05)	5.18(5.04,5.32)	9.32(9.14,9.5)
Egypt	858.82 (618.52-1133.68)	836.29 (614.94-1140.4)	886.81 (648.24-1201.81)	-2.62(-2.72,-2.52)	6.04(5.89,6.19)	3.26(3.15,3.37)
Iran (Islamic Republic of)	1102.97 (869.11-1375.42)	1059.45 (835.93-1321.91)	1064.82 (843.01-1327.57)	-3.95(-4.07,-3.83)	0.51(0.47,0.55)	-3.46(-3.57,-3.35)
Iraq	990.21 (746.73-1277.31)	923.36 (698.61-1200.64)	941.82 (715.16-1230.5)	-6.75(-6.91,-6.59)	2(1.91,2.09)	-4.89(-5.02,-4.76)
Jordan	1185.77 (897.83-1555.16)	1163.46 (894.14-1511.08)	1196.04 (922.7-1533.31)	-1.88(-1.96,-1.8)	2.8(2.7,2.9)	0.87(0.81,0.93)
Kuwait	1118.83 (858.88-1441.45)	1167.88 (899.89-1529.73)	1140.24 (872.55-1471.22)	4.38(4.25,4.51)	-2.37(-2.46,-2.28)	1.91(1.83,1.99)
Lebanon	957.39 (733.89-1243.46)	967.29 (740.97-1262.2)	1046.42 (800.84-1342.95)	1.03(0.97,1.09)	8.18(8.01,8.35)	9.3(9.12,9.48)
Libya	939.03 (732.7-1231.72)	1067.64 (818.52-1391.32)	1067.29 (815.27-1391.25)	13.7(13.49,13.91)	-0.03(-0.04,-0.02)	13.66(13.45,13.87)
Morocco	967.78 (736.85-1243.71)	970.14 (736.26-1248.53)	953.03 (718.6-1249.18)	0.24(0.21,0.27)	-1.76(-1.84,-1.68)	-1.52(-1.6,-1.44)
Oman	690.66 (565.83-844.29)	851.59 (645.65-1093.95)	883.46 (683.02-1126.94)	23.3(23.04,23.56)	3.74(3.62,3.86)	27.92(27.64,28.2)
Palestine	961.64 (734.03-1235.97)	921.5 (706.3-1196.86)	953.75 (724.49-1235.23)	-4.17(-4.29,-4.05)	3.5(3.39,3.61)	-0.82(-0.88,-0.76)
Pakistan	413.2 (331.09-514.74)	411.66 (333.23-515.05)	418.44 (338.22-522.13)	-0.37(-0.41,-0.33)	1.65(1.57,1.73)	1.27(1.2,1.34)
Qatar	1038.81 (851.67-1238.53)	1121.26 (933.09-1335.05)	935.79 (712.87-1191.11)	7.94(7.77,8.11)	-16.54(-16.77,-16.31)	-9.92(-10.11,-9.73)
Saudi Arabia	735.72 (558.04-964.83)	859.36 (655.06-1104.35)	885.4 (674.9-1136.69)	16.81(16.58,17.04)	3.03(2.92,3.14)	20.34(20.09,20.59)
Somalia	887.49 (670.14-1172.75)	924.97 (693.83-1240.44)	948.74 (705.74-1279.51)	4.22(4.1,4.34)	2.57(2.47,2.67)	6.9(6.74,7.06)
Sudan	825.37 (624.94-1072.05)	838.43 (641.1-1081.95)	935.12 (718.35-1231.58)	1.58(1.5,1.66)	11.53(11.33,11.73)	13.3(13.09,13.51)
Syrian Arab Republic	816.73 (622.18-1051.32)	851.19 (649.98-1082.77)	894.55 (683.78-1151.53)	4.22(4.1,4.34)	5.09(4.95,5.23)	9.53(9.35,9.71)
Tunisia	931.6 (697.22-1217.28)	947.8 (718.3-1234.38)	969.3 (740.13-1248.65)	1.74(1.66,1.82)	2.27(2.18,2.36)	4.05(3.93,4.17)
United Arab Emirates	936.63 (724.04-1228.44)	880.04 (677.68-1149.13)	977.39 (737.32-1269.88)	-6.04(-6.19,-5.89)	11.06(10.87,11.25)	4.35(4.22,4.48)
Yemen	849.17 (649.24-1105.29)	845.39 (644.31-1109.06)	902.16 (689.59-1193.22)	-0.45(-0.49,-0.41)	6.72(6.56,6.88)	6.24(6.09,6.39)

EMR=Eastern Mediterranean Region.

Table S2: Age-standardised prevalence rate (per 100,000 persons) of heart failure for females in 1990,2005 and 2019, and their relative percentage change by EMR countries.

	Prevalence rate			%Δ		
	**1990**	**2005**	**2019**	**1990-2005**	**2005-2019**	**1990-2019**
Global	685.99 (561.61-834.72)	653.37 (545.36-787.26)	646.15 (537.36-783.01)	-4.76(-4.89,-4.63)	-1.11(-1.17,-1.05)	-5.81(-5.95,-5.67)
EMR	549.08 (433.77-693.95)	565.94 (448.57-710.37)	582.58 (459.9-729.57)	3.07(2.96,3.18)	2.94(2.84,3.04)	6.1(5.95,6.25)
Afghanistan	474.52 (363.73-615.58)	495.64 (381.8-648.28)	522.3 (400.24-677.49)	4.45(4.32,4.58)	5.38(5.24,5.52)	10.07(9.88,10.26)
Bahrain	694.71 (540.74-895.94)	623.83 (482.88-803.04)	604.47 (470.85-782.2)	-10.2(-10.39,-10.01)	-3.1(-3.21,-2.99)	-12.99(-13.2,-12.78)
Djibouti	643.02 (477.91-855.06)	667.79 (499.35-892.17)	692.48 (516.37-920.5)	3.85(3.73,3.97)	3.7(3.58,3.82)	7.69(7.52,7.86)
Egypt	579.3 (423.39-781.78)	573.18 (422.58-781.56)	607.47 (451.32-825.35)	-1.06(-1.12,-1)	5.98(5.83,6.13)	4.86(4.73,4.99)
Iran (Islamic Republic of)	752.76 (599.75-935.82)	723.14 (579.83-904.51)	717.63 (574.01-894.31)	-3.93(-4.05,-3.81)	-0.76(-0.81,-0.71)	-4.67(-4.8,-4.54)
Iraq	702.07 (539.57-909.14)	670.52 (509.25-859.44)	683.94 (519.92-890.38)	-4.49(-4.62,-4.36)	2(1.91,2.09)	-2.58(-2.68,-2.48)
Jordan	686.22(537.04-882.04)	692.62 (538.54-905.12)	685.42 (530.86-888.29)	0.93(0.87,0.99)	-1.04(-1.1,-0.98)	-0.12(-0.14,-0.1)
Kuwait	778 (599.11-1000.67)	793.67 (623-1028.86)	777.44 (608.33-998.35)	2.01(1.92,2.1)	-2.04(-2.13,-1.95)	-0.07(-0.09,-0.05)
Lebanon	703.87 (542.21-905.29)	708.48 (545.08-914.08)	763.07 (591.02-993.42)	0.65(0.6,0.7)	7.71(7.54,7.88)	8.41(8.24,8.58)
Libya	630.84 (483.24-808.01)	701.13 (538.81-913.73)	710.23 (544.86-921.31)	11.14(10.94,11.34)	1.3(1.23,1.37)	12.58(12.37,12.79)
Morocco	612.1 (466.85-795.16)	624.73 (479.18-807.31)	605.63 (464.01-788.64)	2.06(1.97,2.15)	-3.06(-3.17,-2.95)	-1.06(-1.12,-1)
Oman	490.38 (396.71-613.93)	597.79 (460.69-769.32)	627.32 (481.96-805.35)	21.9(21.64,22.16)	4.94(4.81,5.07)	27.93(27.65,28.21)
Palestine	657.01 (506.58-858.44)	656.61 (506.26-860.82)	669.97 (518.72-864.53)	-0.06(-0.08,-0.04)	2.03(1.94,2.12)	1.97(1.88,2.06)
Pakistan	376.54 (300.4-471.85)	391.48 (312.59-497.1)	391.55 (313.08-490.54)	3.97(3.85,4.09)	0.02(0.01,0.03)	3.99(3.87,4.11)
Qatar	578.34 (460.91-720.71)	578.55 (466.59-717.78)	517.82 (407.46-667.64)	0.04(0.03,0.05)	-10.5(-10.69,-10.31)	-10.46(-10.65,-10.27)
Saudi Arabia	526.73 (410.4-692.67)	612.64 (475.21-779.62)	622.5 (482.24-796.62)	16.31(16.08,16.54)	1.61(1.53,1.69)	18.18(17.94,18.42)
Somalia	542.44 (396.91-733.79)	560.9 (420.46-751.23)	564.43 (417.27-760.67)	3.4(3.29,3.51)	0.63(0.58,0.68)	4.05(3.93,4.17)
Sudan	528.91 (405.39-701.28)	547.41 (424.51-698.65)	610.26 (463.01-783.91)	3.5(3.39,3.61)	11.48(11.28,11.68)	15.38(15.16,15.6)
Syrian Arab Republic	569.9 (432.54-725.82)	603.37 (464.04-769.59)	633.53 (484.32-807.03)	5.87(5.72,6.02)	5(4.86,5.14)	11.17(10.97,11.37)
Tunisia	665.4 (516.32-860.29)	683.55 (528.3-878.53)	692.58 (542.41-893.55)	2.73(2.63,2.83)	1.32(1.25,1.39)	4.08(3.96,4.2)
United Arab Emirates	584.89 (457.61-751.23)	566.34 (440.75-739.94)	608.51 (466.41-795.5)	-3.17(-3.28,-3.06)	7.45(7.29,7.61)	4.04(3.92,4.16)
Yemen	556.73 (432.8-734.82)	567.33 (433.68-741.16)	602.97 (466.29-800.42)	1.9(1.82,1.98)	6.28(6.13,6.43)	8.31(8.14,8.48)

EMR=Eastern Mediterranean Region.

Table S3: Age-standardised YLD rate (per 100,000 persons) of heart failure for males in 1990, 2005 and 2019, and their relative percentage change by EMR countries.

	YLD rate			%Δ		
	**1990**	**2005**	**2019**	**1990-2005**	**2005-2019**	**1990-2019**
Global	77.38 (50.15-111.98)	72.27 (46.98-103.67)	70.58 (45.81-101.35)	-6.6(-6.75,-6.45)	-2.34(-2.43,-2.25)	-8.79(-8.97,-8.61)
EMR	67.01 (42.12-98.16)	70.52 (43.99-102.88)	73.98 (46.52-107.71)	5.24(5.1,5.38)	4.91(4.78,5.04)	10.4(10.21,10.59)
Afghanistan	66.54 (41.53-100.17)	69.31 (43.89-102.9)	74.69 (46.85-111.76)	4.16(4.04,4.28)	7.76(7.59,7.93)	12.25(12.05,12.45)
Bahrain	91.57 (57.32-134.64)	83.11 (52.41-122.26)	82.35 (51.54-120)	-9.24(-9.42,-9.06)	-0.91(-0.97,-0.85)	-10.07(-10.26,-9.88)
Djibouti	93.26 (57.34-137.11)	97.11 (61-144.16)	102.37 (62.65-152.8)	4.13(4.01,4.25)	5.42(5.28,5.56)	9.77(9.59,9.95)
Egypt	77.11 (47.15-117.59)	75.32 (44.99-115.43)	80.02 (48.47-120.86)	-2.32(-2.41,-2.23)	6.24(6.09,6.39)	3.77(3.65,3.89)
Iran (Islamic Republic of)	98.79 (62.8-143.36)	95.27 (60.03-138.1)	96.01 (60.03-139.48)	-3.56(-3.67,-3.45)	0.78(0.73,0.83)	-2.81(-2.91,-2.71)
Iraq	88.8 (54.39-130.13)	83.08 (51.14-121.59)	85.11 (53.25-126.67)	-6.44(-6.59,-6.29)	2.44(2.34,2.54)	-4.16(-4.28,-4.04)
Jordan	106.76 (66.42-157.86)	105.06 (66.98-156.09)	108.29 (68.72-158.17)	-1.59(-1.67,-1.51)	3.07(2.96,3.18)	1.43(1.36,1.5)
Kuwait	100.8 (62.98-149.25)	105.51 (65.52-154.91)	103.2 (65.31-152.55)	4.67(4.54,4.8)	-2.19(-2.28,-2.1)	2.38(2.29,2.47)
Lebanon	86.13 (55.07-127.55)	87.31 (54.51-130.96)	94.63 (59.58-140.14)	1.37(1.3,1.44)	8.38(8.21,8.55)	9.87(9.69,10.05)
Libya	84.37 (53.11-121.5)	96.2 (60.03-140.35)	96.3 (59.51-141.71)	14.02(13.8,14.24)	0.1(0.08,0.12)	14.14(13.92,14.36)
Morocco	87.09 (54.2-129.14)	87.45 (54.77-130.22)	86.15 (52.54-125.5)	0.41(0.37,0.45)	-1.49(-1.57,-1.41)	-1.08(-1.14,-1.02)
Oman	61.75 (39.97-88.74)	76.32 (47.44-112.9)	79.48 (49.94-115.66)	23.6(23.34,23.86)	4.14(4.02,4.26)	28.71(28.43,28.99)
Palestine	86.48 (54.48-127.63)	83.02 (52.67-121.86)	86.2 (55.13-127.54)	-4(-4.12,-3.88)	3.83(3.71,3.95)	-0.32(-0.36,-0.28)
Pakistan	36.43 (23.03-52.77)	36.29 (23.36-52.85)	37.02 (23.56-54.61)	-0.38(-0.42,-0.34)	2.01(1.92,2.1)	1.62(1.54,1.7)
Qatar	93.68 (62.17-133.32)	101.43 (66.82-144.1)	84.8 (54.32-126.2)	8.27(8.1,8.44)	-16.4(-16.63,-16.17)	-9.48(-9.66,-9.3)
Saudi Arabia	65.64 (41.02-97.42)	77.04 (48.41-113.3)	79.8 (49.24-115.77)	17.37(17.14,17.6)	3.58(3.46,3.7)	21.57(21.32,21.82)
Somalia	78.82 (47.37-117.56)	82.18 (51.54-120.74)	84.43 (52.01-126.46)	4.26(4.13,4.39)	2.74(2.64,2.84)	7.12(6.96,7.28)
Sudan	73.83 (45.54-109.57)	75.17 (46.99-111.89)	84.1 (52.16-124.62)	1.81(1.73,1.89)	11.88(11.68,12.08)	13.91(13.7,14.12)
Syrian Arab Republic	73.36 (46.27-107.48)	76.73 (48.04-113.93)	80.76 (50.56-119.4)	4.59(4.46,4.72)	5.25(5.11,5.39)	10.09(9.9,10.28)
Tunisia	83.82 (50.99-123.44)	85.56 (53.42-126.32)	87.67 (54.92-129.1)	2.08(1.99,2.17)	2.47(2.37,2.57)	4.59(4.46,4.72)
United Arab Emirates	84.32 (53.69-124.89)	79.46 (50.41-117.88)	88.4 (55.08-131.2)	-5.76(-5.9,-5.62)	11.25(11.05,11.45)	4.84(4.71,4.97)
Yemen	76.06 (47.01-111.33)	76.04 (46.59-114.36)	81.23 (50.34-120.08)	-0.03(-0.04,-0.02)	6.83(6.67,6.99)	6.8(6.64,6.96)

YLD= years lived with disability. EMR=Eastern Mediterranean Region.

Table S4: Age-standardised YLD rate (per 100,000 persons) of heart failure for females in 1990,2005 and 2019, and their relative percentage change by EMR countries.

	YLD rate			%Δ		
	**1990**	**2005**	**2019**	**1990-2005**	**2005-2019**	**1990-2019**
Global	61.44 (39.63-88.87)	58.58 (38.39-84.17)	57.99 (37.72-83.46)	-4.65(-4.78,-4.52)	-1.01(-1.07,-0.95)	-5.62(-5.76,-5.48)
EMR	48.83 (30.8-71.3)	50.52 (31.87-73.5)	52.21 (33.02-76.47)	3.46(3.35,3.57)	3.35(3.24,3.46)	6.92(6.76,7.08)
Afghanistan	41.99 (26.51-61.78)	43.88 (27.82-64.74)	46.49 (29.46-68.94)	4.5(4.37,4.63)	5.95(5.8,6.1)	10.72(10.53,10.91)
Bahrain	62.23 (39.19-90.82)	56.14 (34.88-81.96)	54.54 (34.83-80.36)	-9.79(-9.97,-9.61)	-2.85(-2.95,-2.75)	-12.36(-12.56,-12.16)
Djibouti	57.25 (35.47-85.78)	59.55 (36.58-90.84)	61.98 (38.16-92.68)	4.02(3.9,4.14)	4.08(3.96,4.2)	8.26(8.09,8.43)
Egypt	51.68 (31.43-77.74)	51.4 (31.08-78.74)	54.64 (33.54-84.37)	-0.54(-0.59,-0.49)	6.3(6.15,6.45)	5.73(5.59,5.87)
Iran (Islamic Republic of)	67.25 (43.07-97.23)	64.91 (41.31-94.16)	64.59 (41.07-93.36)	-3.48(-3.59,-3.37)	-0.49(-0.53,-0.45)	-3.96(-4.08,-3.84)
Iraq	62.58 (39.26-91.57)	60.07 (37.52-89.3)	61.53 (38.51-91.01)	-4.01(-4.13,-3.89)	2.43(2.33,2.53)	-1.68(-1.76,-1.6)
Jordan	61.48 (38.76-90.71)	62.29 (39.18-91.5)	61.86 (39.5-90.47)	1.32(1.25,1.39)	-0.69(-0.74,-0.64)	0.62(0.57,0.67)
Kuwait	70 (43.61-103.34)	71.61 (45.48-105.06)	70.29 (44.11-104.49)	2.3(2.21,2.39)	-1.84(-1.92,-1.76)	0.41(0.37,0.45)
Lebanon	63.03 (39.76-91.93)	63.79 (39.13-92.76)	68.81 (42.93-101.88)	1.21(1.14,1.28)	7.87(7.7,8.04)	9.17(8.99,9.35)
Libya	56.41 (36.36-82.13)	62.93 (39.71-92.21)	63.83 (40.51-94.21)	11.56(11.36,11.76)	1.43(1.36,1.5)	13.15(12.94,13.36)
Morocco	54.77 (34.52-80.59)	56.04 (34.88-82.17)	54.47 (34.54-80.08)	2.32(2.23,2.41)	-2.8(-2.9,-2.7)	-0.55(-0.6,-0.5)
Oman	43.61 (27.83-62.32)	53.33 (33.24-77.48)	56.15 (35.06-81.23)	22.29(22.03,22.55)	5.29(5.15,5.43)	28.75(28.47,29.03)
Palestine	58.7 (36.31-85.66)	58.83 (37.27-85.55)	60.33 (38.41-88.3)	0.22(0.19,0.25)	2.55(2.45,2.65)	2.78(2.68,2.88)
Pakistan	33.05 (21.13-47.92)	34.37 (22.31-49.96)	34.56 (21.9-51)	3.99(3.87,4.11)	0.55(0.5,0.6)	4.57(4.44,4.7)
Qatar	51.84 (32.81-73.94)	52.16 (33.12-74.27)	46.78 (29.6-68.26)	0.62(0.57,0.67)	-10.31(-10.5,-10.12)	-9.76(-9.94,-9.58)
Saudi Arabia	46.58 (30.34-68.8)	54.64 (34.84-79.15)	55.9 (34.69-82.79)	17.3(17.07,17.53)	2.31(2.22,2.4)	20.01(19.76,20.26)
Somalia	48.07 (29.51-72.42)	49.71 (30.77-73.7)	50.12 (30.61-75.3)	3.41(3.3,3.52)	0.82(0.76,0.88)	4.26(4.13,4.39)
Sudan	46.97 (28.82-68.6)	48.81 (30.77-71.4)	54.66 (33.95-80.36)	3.92(3.8,4.04)	11.99(11.79,12.19)	16.37(16.14,16.6)
Syrian Arab Republic	50.93 (31.62-75.67)	54.22 (33.45-80.6)	57.04 (36.02-84.65)	6.46(6.31,6.61)	5.2(5.06,5.34)	12(11.8,12.2)
Tunisia	59.79 (37.43-88.79)	61.62 (39.24-90.98)	62.57 (39.64-92.51)	3.06(2.95,3.17)	1.54(1.46,1.62)	4.65(4.52,4.78)
United Arab Emirates	52.38 (33.21-76.89)	50.99 (32.63-74.39)	54.91 (35.16-80.82)	-2.65(-2.75,-2.55)	7.69(7.52,7.86)	4.83(4.7,4.96)
Yemen	49.6 (31.11-74.01)	50.72 (31.81-74.22)	53.95 (33.63-79.62)	2.26(2.17,2.35)	6.37(6.22,6.52)	8.77(8.59,8.95)

YLD= years lived with disability. EMR=Eastern Mediterranean Region.

Table S5. Age-standardised prevalence rate (per 100,000 persons) of heart failure due to each underlying cause for males in 1990 and 2019 by EMR countries.

	Ischemic heart disease	Hypertensive heart disease	Non-rheumatic valvular heart disease	Rheumatic heart disease	Alcoholic cardiomyopathy
	**1990**				
Global	366.89 (271.04-474.04)	238.87 (172.47-324.37)	33.17 (21.94-48)	19.49 (15.19-24.8)	19.16 (14.62-25.27)
EMR	367.65 (267.13-485.93)	268.44 (193.92-372.64)	3.93 (2.54-5.95)	18.48 (14.2-24.09)	2.03 (1.46-2.84)
Afghanistan	286.67 (205.13-387.88)	388.91 (279.62-538.84)	3.49 (2.18-5.42)	14.72 (10.79-19.8)	1.27 (0.91-1.71)
Bahrain	704.09 (520.4-922.02)	115.3 (82.38-159.36)	7.99 (4.86-12.55)	9.34 (6.86-12.24-)	6.55 (4.79-8.72)
Djibouti	233.06 (151.03-345.42)	356.66 (240.21-513.07)	2.22 (1.39-3.5)	1.2 (0.73-1.87)	2.27 (1.43-3.37)
Egypt	477.51 (317.42-676.28)	288.26 (185.74-436.14-)	5.32 (3.4-8.24)	8.54 (5.68-12.43)	3.17 (2.03-5.09)
Iran (Islamic Republic of)	519.75 (380.89-682.87)	470.53 (334.78-640.14)	4.22 (2.78-6.14)	15.66 (11.7-20.99)	3.43 (2.47-4.65)
Iraq	598.97 (426.98-790.01)	257.5 (183.45-358.53)	6.42 (4.22-9.58)	15.33 (11.28-20.18)	0.71 (0.55-0.92)
Jordan	475.32 (336.52-636.28)	628.41 (456.07-858.1)	4.63 (2.76-7.47)	5.7 (4.23-7.51)	1.17 (0.87-1.55)
Kuwait	517.23 (378.43-690.68)	522.73 (370.17-717.58)	10.76 (6.56-16.47)	5.78 (4.28-7.52)	1.29 (0.96-1.71)
Lebanon	351.48 (250.96-475.08)	498.93 (359.37-680.62)	5.04 (3.2-7.72)	9.7 (7.06-12.78)	4.59 (3.25-6.38)
Libya	429.17 (307.6-581.82)	417.19 (302.85-580.22)	6.45 (4.04-9.9)	7.12 (5.20-9.59)	2.74 (1.97-3.82)
Morocco	476.66 (349.16-634.77)	405.44 (290.61-559.19)	4.13 (2.61-6.5)	13.25 (9.70-17.42)	2.6 (1.87-3.63)
Oman	442.49 (348.53-547.95)	175.93 (134.95-230.25)	6.37 (4.01-10.15)	2.66 (2.04-3.40)	1.26 (0.94-1.65)
Palestine	491.73 (356.81-663.48)	361.36 (260-500.41)	3.61 (2.17-5.86)	5.65 (4.19-7.33)	0.78 (0.59-1)
Pakistan	173.89 (124.84-234.78)	119.05 (82.73-170.22)	2.2 (1.43-3.29)	31.13 (23.69-40.72)	0.86 (0.58-1.23)
Qatar	757.36 (589.26-925.22)	104.96 (79.73-137.46)	18.86 (11.28-29.75)	12.81 (9.99-16.35)	4.41 (3.45-5.59)
Saudi Arabia	515.48 (372.67-695.26)	64.83 (46.69-89.48)	6.43 (4.05-10.28)	8.36 (6.12-11)	0.78 (0.57-1.08)
Somalia	199.91 (129.4-298.11)	296.4 (200.85-422.25)	1.21 (0.79-1.85)	0.98 (0.60-1.53)	2.33 (1.47-3.55)
Sudan	367.32 (264.29-500.86)	378.41 (269.86-523.69)	3.53 (2.16-5.59)	12.06 (8.91-16.14)	1.88 (1.33-2.64)
Syrian Arab Republic	605.44 (441.63-791.43)	108.49 (77.97-148.37)	8.11 (5.81-11.66)	21.06 (15.47-28.03)	2.68 (1.9-3.75)
Tunisia	454.89 (323.05-603.28)	386.81 (276.66-537.18)	4.41 (2.78-6.83)	8.62 (6.24-11.4)	2.5 (1.79-3.43)
United Arab Emirates	367.19 (260.77-507.94)	458.31 (330.3-621.11)	10.24 (6.36-16.37)	17.66 (12.78-23.85)	2.53 (1.84-3.55)
Yemen	362.26 (258.02-496.02)	407.74 (289.59-569.67)	3.81 (2.36-6.05)	14.31 (10.47-19.43)	1.6 (1.13-2.22)
	**2019**				
Global	316.12 (236.51-411.4)	241.25 (176.26-324.85)	28.05 (18.36-41.03)	20.6 (15.98-26.12)	13.33 (10.32-17.25)
EMR	410.74 (298.75-539.64)	299.9 (215.31-414.45)	4.27 (2.77-6.46)	17.65 (13.35-23.14)	2.19 (1.59-3.04)
Afghanistan	335.14 (240.33-453.92)	422.92 (306.05-588.35)	3.36 (2.08-5.19)	16.03 (11.72-21.26)	1.4 (1.01-1.88)
Bahrain	611.12 (446.82-819.78)	109.29 (78.93-149.41)	8.1 (4.98-12.22)	8.95 (6.57-11.79)	6.43 (4.74-8.45)
Djibouti	272.94 (177.23-404.76)	376.11 (253.95-536.45)	1.86 (1.21-2.86)	1.32 (0.83-2.06)	2.52 (1.61-3.68)
Egypt	487.1 (326.46-691.64)	296.45 (189.17-443.97)	5.85 (3.77-9.11)	9.07 (6-13.15)	3.59 (2.25-5.64)
Iran (Islamic Republic of)	494.88 (363.66-649.43)	456.73 (326.82-623.14)	3.74 (2.45-5.45)	16.13 (12.13-21.55)	3.49 (2.52-4.73)
Iraq	557.38 (401.24-746.17)	253.12 (182.98-353.41)	5.33 (3.53-7.97)	15.57 (11.48-20.34)	0.71 (0.55-0.90)
Jordan	453.76 (324.91-606.47)	657.07 (477.4-879.74)	5.34 (3.26-8.13)	5.95 (4.41-7.75)	1.21 (0.89-1.59)
Kuwait	540.38 (389.43-719.41)	519.93 (368.74-715.42)	9.36 (5.61-14.1)	5.77 (4.27-7.58)	1.34 (0.99-1.78)
Lebanon	404.57 (286.89-556.04)	526.53 (374.91-724.2)	3.71 (2.31-5.69)	10.55 (7.76-13.84)	5.07 (3.60-7)
Libya	527.61 (381-707)	436.98 (314.35-604.58)	5.21 (3.24-8.08)	7.77 (5.61-10.28)	3.20 (2.26-4.45)
Morocco	456.34 (327.25-618.69)	408.56 (290.28-570.75)	4.4 (2.8-6.84)	13.36 (9.8-17.57)	2.68 (1.93-3.70)
Oman	592.96 (442.24-766.88)	209.51 (149.21-289.07)	4.89 (3.08-7.41)	3.24 (2.35-4.32)	1.31 (0.93-1.86)
Palestine	486.23 (353.12-654.1)	359.43 (252.87-503.4)	3.65 (2.21-5.93)	5.7 (4.24-7.4)	0.8 (0.61-1.04)

Pakistan	180.8 (129.67-244.69)	119.62 (83.3-169.95)	2.32 (1.51-3.47)	32.22 (24.33-42.32)	0.90 (0.62-1.28)
Qatar	681.41 (497.94-895.73)	98.34 (70.8-133.99)	13.1 (7.99-19.65)	10.53 (7.75-13.49)	3.78 (2.79-4.95)
Saudi Arabia	635.46 (464.84-832.12)	70.69 (50.32-98.06)	7.53 (4.61-11.45)	9.28 (6.78-12.23)	0.93 (0.67-1.28)
Somalia	227.53 (147.72-333.55)	306.82 (205.74-440.37)	0.98 (0.65-1.47)	1.04 (0.65-1.64)	2.53 (1.59-3.75)
Sudan	438.34 (311.6-595.76)	411.82 (294.17-573.47)	3.8 (2.4-5.92)	13.66 (10.13-18.32)	2.04 (1.44-2.86)
Syrian Arab Republic	672.28 (496.71-878.84)	113.83 (81.08-159.84)	6.5 (4.87-8.79)	22.61 (16.68-30.1)	3.03 (2.13-4.28)
Tunisia	479.58 (345.36-646.41)	394.15 (282.19-547.67)	3.93 (2.50-5.93)	8.96 (6.63-11.81)	2.74 (1.98-3.78)
United Arab Emirates	394.82 (280.23-531.91)	465.17 (329.87-638.01)	13.36 (8.23-20.45)	17.39 (12.4-23.65)	2.42 (1.72-3.32)
Yemen	397.45 (286.15-535.64)	424.1 (302.33-587.25)	3.56 (2.26-5.55)	15.18 (11.11-20.05)	1.69 (1.21-2.32)

EMR=Eastern Mediterranean Region.

Table S6. Age-standardised prevalence rate (per 100,000 persons) of heart failure due to each underlying cause for females in 1990 and 2019 by EMR countries.

	Ischemic heart disease	Hypertensive heart disease	Non-rheumatic valvular heart disease	Rheumatic heart disease	Alcoholic cardiomyopathy
	**1990**				
Global	257.86 (189.94-335.7)	202.19 (147.16-277.09)	47.42 (31.42-68.53)	28.87 (22.15-37.07)	6.86 (5.19-9.05)
EMR	222.48 (161.19-298.87)	245.54 (178.54-333.47)	5.23 (3.50-7.88)	22.1 (16.63-29.04)	1.17 (0.84-1.62)
Afghanistan	153.86 (109-206.48)	271.23 (196.05-371.53)	5.27 (3.30-8.28)	13.26 (9.60-17.92)	1.68 (1.15-2.32)
Bahrain	358.79 (258-488.11)	206.38 (148.01-286.08)	11.03 (7-17.43)	14.17 (10.56-19.05)	2.99 (2.23-4.03)
Djibouti	107.26 (68.92-159.44)	251.48 (164.92-357.39)	2.38 (1.54-3.65)	3.63 (2.35-5.53)	1.05 (0.67-1.53)
Egypt	256.86 (169.42-377.81)	254.38 (169.11-369.35)	6.70 (4.37-10.32)	9.19 (6.13-13.43)	1.68 (1.05-2.58)
Iran (Islamic Republic of)	297.89 (219.77-395.42)	371.82 (271.47-491.78)	6.01 (4-8.72)	19.09 (13.97-26.14)	1.05 (0.79-1.37)
Iraq	368.18 (266.4-496.11)	247.92 (178.07-346.24)	8.6 (5.55-13.49)	15.23 (11.13-20.32)	0.75 (0.55-1.05)
Jordan	178.87 (131.26-238.3)	449.18 (332.42-599.39)	6.09 (3.76-9.61)	5.62 (4.07-7.52)	0.37 (0.27-0.48)
Kuwait	205.12 (145.86-277.77)	502.97 (368.14-665.77)	8.21 (5.14-12.62)	7.44 (5.41-9.91)	0.7 (0.50-0.94)
Lebanon	305.02 (218.09-407.26)	328.86 (235.29-449.72)	6.75 (4.41-10.33)	7.56 (5.54-10.14)	1.06 (0.78-1.45)
Libya	245.13 (174.93-329.14)	321.01 (232.96-433.71)	7.55 (4.76-11.80)	9.20 (6.80-12.12)	0.91 (0.66-1.28)
Morocco	247.71 (177.2-335.84)	302.85 (218.64-415.13)	6.03 (3.82-9.23)	14.12 (10.29-18.86)	1.75 (1.22-2.53)
Oman	244.19 (183.76-317.32)	189.29 (140-250.86)	8.69 (5.52-13.78)	3.35 (2.52-4.34)	0.43 (0.32-0.58)
Palestine	264.31 (188.14-357.01)	315.5 (230.89-432.88)	5.05 (2.95-8.27)	8.55 (6.19-11.41)	1.41 (1.02-1.93)
Pakistan	131.77 (92.08-183.99)	151.79 (105.2-212.57)	1.73 (1.15-2.54)	42.94 (31.94-56.75)	0.45 (0.30-0.67)
Qatar	344.29 (257.47-449.57)	135.62 (101.26-185.86)	13.74 (8.51-21.75)	9.79 (7.41-12.84)	1.8 (1.37-2.34)
Saudi Arabia	289.56 (207.82-394.4)	119.15 (84.41-161.23)	10.75 (6.68-17.39)	11.17 (8.13-15.13)	1.98 (1.39-2.76)
Somalia	92.68 (59.92-139.58)	212.58 (140.21-313.25)	1.54 (1-2.28)	3.01 (1.91-4.72)	3.13 (1.96-4.68)
Sudan	196.57 (138.89-268.14)	279.44 (200.06-385.19)	5.22 (3.32-8.07)	12.02 (8.83-16.34)	2.1 (1.43-3.02)
Syrian Arab Republic	380.3 (277.88-503.5)	103.5 (74.44-144.48)	14.35 (10.86-19.60)	21.26 (15.44-28.36)	1.31 (0.89-1.83)
Tunisia	243.51 (173.3-330.4)	353.84 (255.58-470.45)	6.20 (3.97-9.63)	10.52 (7.7-14.02)	0.92 (0.67-1.26)
United Arab Emirates	184.87 (131.34-255.36)	322.03 (233.78-436.37)	11.20 (7.16-17.49)	13.76 (10.15-18.56)	1.17 (0.84-1.58)
Yemen	188.32 (132.6-262.49)	311.19 (229.7-425.3)	5.53 (3.50-8.48)	13.13 (9.55-17.65)	1.40 (0.99-2)
	**2019**				
Global	222.48 (163.75-291.22)	224.60 (162.85-302.06)	33.67 (22.07-49.98)	31.59 (24.49-40.34)	4.13 (3.18-5.36)
EMR	238 (172.13-321.01)	262.32 (192.72-354.69)	5.31 (3.55-7.76)	22 (16.69-28.86)	1.20 (0.86-1.65)
Afghanistan	173.22 (123.05-235.32)	297.18 (214.17-403.54)	5.21 (3.24-8.21)	14.58 (10.62-19.64)	1.78 (1.21-2.46)
Bahrain	284.57 (201.77-386.43)	197.82 (142.19-272.88)	9.18 (5.87-13.93)	13.53 (9.96-18.06)	2.91 (2.14-3.94)

Djibouti	121.68 (75.7-180.89)	261.76 (172.65-379.83)	1.96 (1.30-2.93)	4 (2.59-6.11)	1.14 (0.71-1.64)
Egypt	267.05 (177.17-383.68)	264.80 (174.45-391.49)	7.36 (4.83-11.11)	10.01 (6.73-14.65)	1.89 (1.20-3.01)
Iran (Islamic Republic of)	279.44 (206.22-370.49)	356.20 (261.62-470.09)	5.51 (3.70-7.91)	18.99 (14.01-25.95)	1.06 (0.80-1.38)
Iraq	353.17 (248.66-479.31)	247.71 (177.04-345.01)	7.26 (4.79-11.08)	15.64 (11.49-21.07)	0.72 (0.52-0.99)
Jordan	166.91 (120.16-223.32)	459.58 (340.5-617.66)	5.70 (3.46-8.93)	5.77 (4.21-7.67)	0.39 (0.29-0.51)
Kuwait	202.97 (147.23-274.18)	503.70 (377.11-665.78)	7.4 (4.59-11.06)	7.51 (5.62-9.96)	0.71 (0.53-0.95)
Lebanon	341.99 (245.18-461.36)	349.02 (249.92-479.11)	5.04 (3.34-7.52)	8.11 (5.98-10.66)	1.15 (0.84-1.54)
Libya	300.23 (213.37-414.44)	339.69 (243.76-465.65)	6.70 (4.33-10.25)	9.93 (7.27-13.38)	1.06 (0.76-1.47)
Morocco	235.72 (167.24-318.97)	306.51 (222.12-418.78)	6.46 (4.17-9.95)	14.29 (10.52-18.96)	1.85 (1.28-2.59)
Oman	329.38 (237.13-434.89)	240.38 (173.31-328.45)	6.58 (4.29-9.92)	4.57 (3.35-6.1)	0.34 (0.24-0.47)
Palestine	272.09 (195.26-364.54)	318.83 (231.91-437)	5.24 (3.09-8.47)	8.79 (6.4-11.83)	1.49 (1.09-2)
Pakistan	140.85 (97.92-195.51)	157.35 (108.77-227.14)	1.77 (1.17-2.60)	45.05 (33.42-59)	0.47 (0.31-0.68)
Qatar	295.99 (215.01-396.08)	129.66 (93.02-179)	11.92 (7.36-18.13)	8.91 (6.55-11.87)	1.68 (1.26-2.23)
Saudi Arabia	361.6 (258.06-491.21)	130.51 (94.79-178.63)	9.77 (6.05-15.13)	12.3 (8.84-16.66)	2.36 (1.65-3.33)
Somalia	101.91 (65.96-152.41)	214.37 (143.71-315.01)	1.22 (0.83-1.83)	3.19 (2.02-4.89)	3.29 (2.10-4.85)
Sudan	245.73 (172.61-330.41)	307.59 (221.37-419.19)	5.77 (3.68-8.98)	13.74 (9.97-18.33)	2.30 (1.56-3.25)
Syrian Arab Republic	432.53 (315.48-565.04)	110.75 (79.11-154.34)	12.97 (10.17-16.86)	23.04 (16.74-31.03)	1.50 (1.05-2.11)
Tunisia	257 (186.18-342.4)	363.62 (267.42-494.66)	5.69 (3.66-8.62)	10.89 (7.95-14.36)	1.05 (0.78-1.38)
United Arab Emirates	200.95 (142.03-270.21)	330.07 (237.55-452.35)	11.64 (7.46-17.80)	13.67 (10.01-18.89)	1.06 (0.76-1.45)
Yemen	215.44 (151.46-296.3)	329.42 (241.37-446.61)	5.34 (3.40-8.20)	14.13 (10.30-18.97)	1.50 (1.04-2.14)

EMR=Eastern Mediterranean Region.

Table S7. Age-standardised YLD rate (per 100,000 persons) of heart failure due to each underlying cause for males in 1990 and 2019 by EMR countries.

	Ischemic heart disease	Hypertensive heart disease	Non-rheumatic valvular heart disease	Rheumatic heart disease	Alcoholic cardiomyopathy
	1990				
Global	32.94 (19.97-49.63)	21.41 (12.72-33.15)	2.99 (1.70-5.04)	1.77 (1.11-2.63)	1.75 (1.09-2.58)
EMR	32.84 (19.89-50.07)	24.01 (14.14-37.55)	0.36 (0.20-0.62)	1.67 (1.04-2.47)	0.19 (0.11-0.28)
Afghanistan	25.55 (15.12-39.57)	34.67 (21.05-55.01)	0.32 (0.17-0.56)	1.34 (0.79-2.05)	0.12 (0.07-0.18)
Bahrain	63.34 (39.12-95.64)	10.4 (6.19-16.19)	0.73 (0.39-1.30)	0.85 (0.52-1.35)	0.60 (0.36-0.93)
Djibouti	20.80 (11.59-34.69)	31.81 (17.78-50.51)	0.21 (0.11-0.36)	0.11 (0.06-0.19)	0.21 (0.12-0.34)
Egypt	42.80 (24.22-67.41)	25.86 (14.04-43.62)	0.49 (0.26-0.87)	0.78 (0.44-1.26)	0.29 (0.16-0.49)
Iran (Islamic Republic of)	46.48 (28.41-71.46)	42.12 (24.48-64.92)	0.39 (0.22-0.63)	1.42 (0.88-2.19)	0.32 (0.19-0.48)
Iraq	53.65 (31.94-82.85)	23.03 (13.41-36.06)	0.59 (0.33-1)	1.39 (0.82-2.12)	0.07 (0.04-0.10)
Jordan	42.75 (25.50-65.83)	56.53 (33.30-88.75)	0.42 (0.22-0.76)	0.53 (0.32-0.81)	0.11 (0.07-0.16)
Kuwait	46.58 (28.29-72.51)	47.03 (27.86-72.32)	0.98 (0.52-1.71)	0.53 (0.33-0.80)	0.12 (0.07-0.18)
Lebanon	31.54 (18.62-48.94)	44.87 (26.73-68.53)	0.46 (0.25-0.80)	0.88 (0.53-1.39)	0.42 (0.26-0.66)
Libya	38.49 (23.08-58.71)	37.46 (22.50-56.73)	0.59 (0.32-1.04)	0.65 (0.39-1.01)	0.25 (0.15-0.40)
Morocco	42.83 (25.79-65.64)	36.47 (21.65-57.27)	0.38 (0.20-0.68)	1.21 (0.73-1.86)	0.24 (0.14-0.37)
Oman	39.48 (24.50-57.39)	15.72 (9.73-23.49)	0.57 (0.30-1.01)	0.25 (0.16-0.37)	0.12 (0.07-0.18)
Palestine	44.19 (27.19-66.67)	32.42 (19.25-50.04)	0.33 (0.17-0.59)	0.52 (0.32-0.80)	0.07 (0.05-0.11)
Pakistan	15.29 (9.01-23.31)	10.47 (5.94-16.97)	0.20 (0.11-0.35)	2.79 (1.72-4.20)	0.08 (0.05-0.13)
Qatar	68.21 (44.29-97.63)	9.45 (5.80-14)	1.70 (0.87-3)	1.18 (0.74-1.81)	0.41 (0.27-0.59)
Saudi Arabia	45.89 (28.15-70.62)	5.77 (3.34-9.35)	0.58 (0.31-1.02)	0.76 (0.45-1.19)	0.07 (0.04-0.11)
Somalia	17.80 (9.80-30.01)	26.36 (14.65-42.57)	0.11 (0.06-0.19)	0.09 (0.05-0.16)	0.22 (0.12-0.35)
Sudan	32.79 (19.57-50.59)	33.82 (20.08-53.25)	0.32 (0.17-0.58)	1.10 (0.66-1.73)	0.17 (0.10-0.27)
Syrian Arab Republic	54.27 (32.94-81.95)	9.75 (5.66-15.26)	0.74 (0.43-1.17)	1.92 (1.14-3.05)	0.25 (0.15-0.39)
Tunisia	40.85 (24.02-61.93)	34.79 (20.33-54.48)	0.40 (0.22-0.69)	0.79 (0.48-1.21)	0.23 (0.14-0.36)
United Arab Emirates	32.99 (19.75-50.36)	41.24 (25.19-63.98)	0.93 (0.50-1.64)	1.60 (0.91-2.57)	0.23 (0.14-0.37)
Yemen	32.41 (18.98-49.51)	36.48 (22.01-56.5)	0.35 (0.19-0.61)	1.30 (0.77-2.02)	0.15 (0.09-0.23)
	2019				
Global	28.43 (17.39-42.37)	21.72 (12.96-33.92)	2.53 (1.41-4.30)	1.87 (1.17-2.80)	1.22 (0.78-1.82)
EMR	36.92 (22.41-56.43)	26.96 (15.81-42.60)	0.39 (0.22-0.67)	1.6 (0.98-2.37)	0.20 (0.12-0.31)
Afghanistan	30 (18.12-45.89)	37.92 (22.93-58.41)	0.31 (0.16-0.54)	1.46 (0.88-2.22)	0.13 (0.08-0.20)
Bahrain	55.21 (34.08-83.18)	9.89 (5.91-15.36)	0.74 (0.39-1.27)	0.82 (0.49-1.30)	0.59 (0.35-0.93)
Djibouti	24.41 (13.39-40.25)	33.71 (18.73-54.38)	0.17 (0.09-0.30)	0.12 (0.06-0.21)	0.23 (0.13-0.37)
Egypt	43.87 (25.69-70.66)	26.75 (14.29-45.27)	0.54 (0.30-0.93)	0.83 (0.45-1.40)	0.33 (0.18-0.56)
Iran (Islamic Republic of)	44.58 (27.28-68.31)	41.15 (24.10-62.94)	0.34 (0.19-0.58)	1.47 (0.91-2.30)	0.32 (0.20-0.50)
Iraq	50.33 (30-79.17)	22.83 (13.25-35.65)	0.49 (0.27-0.83)	1.42 (0.83-2.16)	0.07 (0.04-0.10)
Jordan	41.05 (24.14-62.95)	59.47 (35.20-91.21)	0.49 (0.26-0.86)	0.55 (0.34-0.85)	0.11 (0.07-0.17)
Kuwait	48.90 (29.27-73.68)	47 (27.63-72.86)	0.85 (0.45-1.50)	0.53 (0.33-0.80)	0.12 (0.08-0.19)
Lebanon	36.52 (21.71-56.94)	47.58 (28.11-72.11)	0.34 (0.18-0.60)	0.96 (0.59-1.47)	0.47 (0.28-0.73)
Libya	47.55 (28.24-73.09)	39.4 (23.17-61.13)	0.48 (0.26-0.84)	0.71 (0.43-1.09)	0.30 (0.18-0.47)
Morocco	41.21 (24.52-63.89)	36.92 (21.57-56.9)	0.40 (0.22-0.71)	1.22 (0.73-1.87)	0.25 (0.15-0.39)
Oman	53.29 (33.1-80.18)	18.82 (11.18-29.14)	0.45 (0.24-0.78)	0.30 (0.18-0.46)	0.12 (0.07-0.19)
Palestine	43.91 (26.87-66.81)	32.43 (18.99-50.12)	0.33 (0.17-0.61)	0.53 (0.33-0.79)	0.07 (0.05-0.11)
Pakistan	15.96 (9.49-24.87)	10.55 (6.02-17.32)	0.21 (0.12-0.37)	2.89 (1.76-4.31)	0.08 (0.05-0.13)
Qatar	61.68 (38.07-94.43)	8.92 (5.29-13.85)	1.19 (0.63-2.05)	0.97 (0.59-1.46)	0.35 (0.21-0.53)

Saudi Arabia	57.2 (34.07-86.61)	6.37 (3.76-10.26)	0.69 (0.37-1.24)	0.85 (0.51-1.33)	0.09 (0.05-0.13)
Somalia	20.25 (11.35-33.27)	27.35 (15.14-44.94)	0.09 (0.05-0.15)	0.10 (0.05-0.17)	0.23 (0.13-0.38)
Sudan	39.39 (23.48-60.57)	37 (22.03-57.70)	0.35 (0.18-0.60)	1.25 (0.75-1.93)	0.19 (0.11-0.29)
Syrian Arab Republic	60.62 (37.29-90.61)	10.26 (5.99-16.11)	0.60 (0.36-0.96)	2.06 (1.24-3.21)	0.28 (0.17-0.44)
Tunisia	43.30 (25.39-66.95)	35.66 (21.24-54.95)	0.36 (0.20-0.64)	0.82 (0.50-1.28)	0.25 (0.15-0.39)
United Arab Emirates	35.64 (21.13-54.65)	42.07 (25.07-65.33)	1.21 (0.63-2.12)	1.59 (0.91-2.52)	0.22 (0.13-0.35)
Yemen	35.72 (21.41-54.6)	38.19 (22.32-58.47)	0.33 (0.17-0.58)	1.38 (0.84-2.10)	0.16 (0.09-0.25)

YLD= years lived with disability. EMR=Eastern Mediterranean Region.

Table S8. Age-standardised YLD rate (per 100,000 persons) of heart failure due to each underlying cause for females in 1990 and 2019 by EMR countries.

	Ischemic heart disease	Hypertensive heart disease	Non-rheumatic valvular heart disease	Rheumatic heart disease	Alcoholic cardiomyopathy
			**1990**		
Global	23.09 (14.07-35.05)	18.09 (10.85-27.87)	4.27 (2.45-7.22)	2.61 (1.62-3.89)	0.63 (0.39-0.94)
EMR	19.72 (11.99-30.03)	21.82 (12.91-33.57)	0.47 (0.27-0.80)	1.99 (1.24-2.98)	0.11 (0.06-0.17)
Afghanistan	13.55 (8.04-20.75)	23.97 (14.49-36.47)	0.47 (0.24-0.83)	1.20 (0.70-1.88)	0.16 (0.09-0.25)
Bahrain	31.99 (19.11-48.84)	18.53 (10.98-28.78)	0.99 (0.52-1.72)	1.29 (0.79-2.02)	0.28 (0.17-0.42)
Djibouti	9.54 (5.18-16.14)	22.4 (12.36-36.11)	0.22 (0.12-0.38)	0.34 (0.19-0.55)	0.10 (0.05-0.16)
Egypt	22.84 (13.01-37.24)	22.66 (12.57-37.01)	0.61 (0.33-1.05)	0.84 (0.49-1.41)	0.16 (0.08-0.26)
Iran (Islamic Republic of)	26.5 (16.12-41.05)	33.21 (19.86-50.70)	0.54 (0.31-0.89)	1.74 (1.06-2.67)	0.10 (0.06-0.15)
Iraq	32.7 (19.72-49.93)	22.08 (13.07-34.43)	0.77 (0.42-1.35)	1.38 (0.82-2.22)	0.07 (0.04-0.11)
Jordan	15.96 (9.50-24.40)	40.19 (24.85-60.41)	0.55 (0.29-0.98)	0.52 (0.32-0.79)	0.03 (0.02-0.05)
Kuwait	18.36 (10.93-28.39)	45.25 (27.04-68.61)	0.74 (0.39-1.34)	0.68 (0.42-1.04)	0.06 (0.04-0.10)
Lebanon	27.22 (16.10-41.49)	29.44 (17.66-45.29)	0.61 (0.33-1.03)	0.70 (0.43-1.05)	0.1 (0.06-0.15)
Libya	21.83 (12.90-33.48)	28.69 (17.05-44.33)	0.68 (0.36-1.20)	0.85 (0.51-1.29)	0.08 (0.05-0.13)
Morocco	22.12 (13.39-33.65)	27.05 (16.08-42.16)	0.55 (0.30-0.94)	1.29 (0.77-2.02)	0.16 (0.09-0.27)
Oman	21.61 (13.19-31.99)	16.83 (10.12-25.57)	0.77 (0.41-1.37)	0.31 (0.19-0.48)	0.04 (0.02-0.06)
Palestine	23.53 (14.21-36.06)	28.13 (16.86-44)	0.46 (0.23-0.84)	0.79 (0.48-1.21)	0.13 (0.08-0.2)
Pakistan	11.52 (6.89-17.71)	13.26 (7.61-21.03)	0.16 (0.09-0.27)	3.83 (2.32-5.80)	0.04 (0.02-0.07)
Qatar	30.71 (18.55-45.47)	12.17 (7.28-18.37)	1.23 (0.66-2.15)	0.90 (0.55-1.39)	0.17 (0.10-0.25)
Saudi Arabia	25.42 (15.53-39.36)	10.57 (6.15-16.59)	0.95 (0.49-1.69)	1.02 (0.60-1.55)	0.18 (0.11-0.29)
Somalia	8.20 (4.50-13.90)	18.86 (10.63-30.34)	0.14 (0.08-0.24)	0.28 (0.15-0.48)	0.29 (0.15-0.48)
Sudan	17.36 (10.45-26.53)	24.8 (14.69-38.41)	0.47 (0.26-0.82)	1.09 (0.67-1.68)	0.19 (0.11-0.31)
Syrian Arab Republic	33.89 (20.45-51.64)	9.24 (5.45-14.45)	1.29 (0.76-2.03)	1.92 (1.09-3)	0.12 (0.07-0.19)
Tunisia	21.79 (13-34.17)	31.79 (19.24-48.62)	0.56 (0.30-1)	0.96 (0.59-1.52)	0.09 (0.05-0.13)
United Arab Emirates	16.48 (9.79-25.05)	28.81 (17.58-44.21)	1.01 (0.52-1.75)	1.25 (0.75-1.94)	0.11 (0.07-0.17)
Yemen	16.72 (9.94-25.69)	27.69 (16.54-42.18)	0.50 (0.27-0.85)	1.19 (0.71-1.83)	0.13 (0.08-0.20)
			**2019**		
Global	19.94 (12.17-29.83)	20.16 (12.17-31.21)	3.04 (1.71-5.10)	2.86 (1.80-4.29)	0.38 (0.24-0.56)
EMR	21.26 (12.93-32.76)	23.49 (13.76-36.48)	0.48 (0.27-0.82)	1.99 (1.22-3.02)	0.11 (0.07-0.17)
Afghanistan	15.34 (9.01-23.40)	26.43 (16.07-41.16)	0.47 (0.25-0.82)	1.32 (0.80-2.11)	0.16 (0.09-0.26)
Bahrain	25.6 (15.28-39.06)	17.83 (10.62-27.79)	0.84 (0.46-1.47)	1.24 (0.73-1.94)	0.27 (0.17-0.42)
Djibouti	10.88 (5.88-18.02)	23.46 (12.66-37.12)	0.18 (0.10-0.31)	0.37 (0.20-0.64)	0.11 (0.06-0.17)
Egypt	23.92 (13.49-39.08)	23.84 (12.88-39.87)	0.67 (0.38-1.17)	0.92 (0.54-1.50)	0.18 (0.10-0.29)

Iran (Islamic Republic of)	25.07 (15.2-38.83)	32.05 (19.02-48.68)	0.50 (0.28-0.81)	1.73 (1.06-2.72)	0.10 (0.06-0.15)
Iraq	31.67 (18.71-49.34)	22.27 (13.18-34.53)	0.66 (0.36-1.15)	1.43 (0.83-2.22)	0.07 (0.04-0.10)
Jordan	15.01 (9.02-23.1)	41.44 (25.77-62.57)	0.52 (0.27-0.93)	0.53 (0.33-0.81)	0.04 (0.02-0.05)
Kuwait	18.30 (11.04-28.20)	45.5 (27.89-68.4)	0.67 (0.36-1.18)	0.69 (0.43-1.05)	0.07 (0.04-0.10)
Lebanon	30.77 (18.54-46.88)	31.46 (18.87-49.15)	0.46 (0.25-0.80)	0.75 (0.46-1.16)	0.11 (0.07-0.16)
Libya	26.87 (16.02-41.09)	30.55 (18.55-47.3)	0.61 (0.33-1.08)	0.91 (0.55-1.38)	0.10 (0.06-0.15)
Morocco	21.12 (12.57-32.46)	27.57 (16.38-42.34)	0.59 (0.31-1.01)	1.3 (0.79-2.07)	0.17 (0.10-0.27)
Oman	29.39 (17.58-44.16)	21.51 (13.03-33.07)	0.59 (0.33-1.04)	0.42 (0.26-0.63)	0.03 (0.02-0.05)
Palestine	24.45 (14.51-36.89)	28.66 (17.05-44.48)	0.47 (0.24-0.87)	0.81 (0.49-1.25)	0.14 (0.09-0.21)
Pakistan	12.38 (7.30-19.36)	13.83 (7.85-22.30)	0.16 (0.09-0.27)	4.03 (2.43-6.09)	0.04 (0.02-0.07)
Qatar	26.61 (15.77-40.15)	11.77 (7.03-18.24)	1.08 (0.56-1.88)	0.82 (0.50-1.25)	0.16 (0.10-0.24)
Saudi Arabia	32.34 (19.11-49.94)	11.75 (7.02-18.44)	0.88 (0.46-1.59)	1.13 (0.68-1.75)	0.22 (0.13-0.34)
Somalia	9.04 (5.02-15.16)	19.06 (10.13-30.96)	0.11 (0.07-0.19)	0.30 (0.16-0.49)	0.3 (0.16-0.48)
Sudan	21.94 (13.22-33.72)	27.53 (16.24-42.92)	0.52 (0.27-0.90)	1.26 (0.74-2)	0.21 (0.12-0.34)
Syrian Arab Republic	38.85 (24-58.29)	9.97 (5.87-15.68)	1.17 (0.70-1.80)	2.10 (1.25-3.37)	0.14 (0.08-0.22)
Tunisia	23.16 (14.24-35.27)	32.83 (19.84-51.12)	0.52 (0.28-0.91)	1 (0.62-1.51)	0.10 (0.06-0.15)
United Arab Emirates	18.03 (10.88-27.73)	29.81 (17.99-45.87)	1.06 (0.57-1.89)	1.24 (0.72-2.05)	0.10 (0.06-0.15)
Yemen	19.19 (11.28-30.58)	29.46 (17.43-45.33)	0.49 (0.26-0.84)	1.29 (0.77-2.01)	0.14 (0.08-0.23)

YLD= years lived with disability. EMR=Eastern Mediterranean Region.
