# Supplementary material for: Changes in the burden and underlying causes of heart failure in the Eastern Mediterranean Region, 1990–2019: An analysis of the Global Burden of Disease Study 2019
Source: eClinicalMedicine. 2022 Dec 26;56:101788. doi: 10.1016/j.eclinm.2022.101788 (PMC9803705; doi:10.1016/j.eclinm.2022.101788)
Supplement: Caption for supplementary material [file mmc2.docx]

**Supplementary material**

**Data sources**

**Figure S1: Age-standardised prevalence rate of heart failure (per 100,000 persons) for males and females in EMR countries in 1990 and 2019.**

**Figure S2: Age-standardised YLD rate of heart failure (per 100,000 persons) for males and females in EMR countries in 1990 and 2019.**

**Figure S3: The HAQ Index and level of SDI for 22 EMR countries in 1990,1995, 2000,2005, 2010 and 2016.**

**Figure S4: Trends in age-standardised prevalence rate of heart failure for 22 EMR countries by SDI, 1990–2019.**

**Figure S5: Trends in age-standardised YLD rate of heart failure for 22 EMR countries by SDI, 1990–2019.**

**Figure S6: Trends in age-standardised prevalence rate of heart failure for 22 EMR countries by HAQ Index in 1990,1995, 2000,2005,2010 and 2016.**

**Figure S7: Trends in age-standardised YLD rate of heart failure for 22 EMR countries by HAQ Index in 1990,1995, 2000,2005,2010 and 2016.**

**Table S1: Age-standardised prevalence rate (per 100,000 persons) of heart failure for males in 1990,2005 and 2019, and their relative percentage change by EMR countries.**

**Table S2: Age-standardised prevalence rate (per 100,000 persons) of heart failure for females in 1990,2005 and 2019, and their relative percentage change by EMR countries.**

**Table S3: Age-standardised YLD rate (per 100,000 persons) of heart failure for males in 1990, 2005 and 2019, and their relative percentage change by EMR countries.**

**Table S4: Age-standardised YLD rate (per 100,000 persons) of heart failure for females in 1990,2005 and 2019, and their relative percentage change by EMR countries.**

**Table S5. Age-standardised prevalence rate (per 100,000 persons) of heart failure due to each underlying cause for males in 1990 and 2019 by EMR countries.**

**Table S6. Age-standardised prevalence rate (per 100,000 persons) of heart failure due to each underlying cause for females in 1990 and 2019 by EMR countries.**

**Table S7. Age-standardised YLD rate (per 100,000 persons) of heart failure due to each underlying cause for males in 1990 and 2019 by EMR countries.**

**Table S8. Age-standardised YLD rate (per 100,000 persons) of heart failure due to each underlying cause for females in 1990 and 2019 by EMR countries.**
